# Towards Greener and More Sustainable Synthesis of MXenes: A Review

**DOI:** 10.3390/nano12234280

**Published:** 2022-12-01

**Authors:** Tahta Amrillah, Che Azurahanim Che Abdullah, Angga Hermawan, Fitri Nur Indah Sari, Vani Novita Alvani

**Affiliations:** 1 Department of Nanotechnology, Faculty of Advanced Technology and Multidiscipline, Universitas Airlangga, Surabaya 60115, East Java, Indonesia; 2 Department of Physics, Faculty of Science, University Putra Malaysia, Serdang 43400, Selangor, Malaysia; 3 Nanomaterial Synthesis and Characterization Laboratory, Institute of Nanoscience and Nanotechnology, Universiti Putra Malaysia, Serdang 43400, Selangor, Malaysia; 4 Research Center for Advanced Materials, National Research and Innovation Agency (BRIN), South Tangerang 15315, Banten, Indonesia; 5 Department of Materials Science and Engineering, National Cheng Kung University, Tainan 70101, Taiwan; 6 Graduate School of Environmental Studies, Tohoku University, Sendai 9808579, Japan

**Keywords:** MXenes, green synthesis, 2D materials, sustainable, biocompatibility

## Abstract

The unique properties of MXenes have been deemed to be of significant interest in various emerging applications. However, MXenes provide a major drawback involving environmentally harmful and toxic substances for its general fabrication in large-scale production and employing a high-temperature solid-state reaction followed by selective etching. Meanwhile, how MXenes are synthesized is essential in directing their end uses. Therefore, making strategic approaches to synthesize greener, safer, more sustainable, and more environmentally friendly MXenes is imperative to commercialize at a competitive price. With increasing reports of green synthesis that promote advanced technologies and non-toxic agents, it is critical to compile, summarize, and synthesize the latest development of the green-related technology of MXenes. We review the recent progress of greener, safer, and more sustainable MXene synthesis with a focus on the fundamental synthetic process, the mechanism, and the general advantages, and the emphasis on the MXene properties inherited from such green synthesis techniques. The emerging use of the so-called green MXenes in energy conversion and storage, environmental remediation, and biomedical applications is presented. Finally, the remaining challenges and prospects of greener MXene synthesis are discussed.

## 1. Introduction

**Figure 1:**
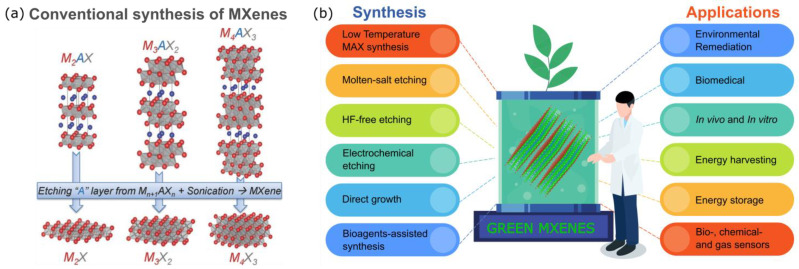
(**a**). Crystal structure transformation of MAX phase and their conventional exfoliation into MXenes. Adopted from reference [3]. (**b**) Green route to synthesize MXenes and their potential applications.

MXenes are a new family of two-dimensional (2D) materials constructed of surface-modified carbide, nitride, and carbonitride. Their structures may vary depending on their chemical composition. Having a general chemical formula of M_n+1_X_n_T_x_ (n = 1, 2, 3, and 4), MXenes are composed of layers of early transition metals (M) which are inserted with n layers of carbon or nitrogen (X) and terminated with surface functional groups (T*_x_* = –O, –OH, and –F) [1,2]. Like other 2D materials, MXenes are conventionally synthesized by exfoliating their initial 3D precursors, namely the MAX phases, which are ternary carbides or nitrides with the general formula of M_n+1_AX_n_ [1,2]. In the MAX phase, as shown in Figure 1a, the M–X bonds are stronger than the M–A bonds. Here, the A layers are chemically more active than the M–X layers are; thus, the A layers are easily removed by etching, mostly by using a strong acid such as hydrofluoric acid (HF) to obtain the M_n+1_X_n_ layers that are typically terminated by fluorine (–F), hydroxide (–OH) and oxygen (–O) groups due to their high surface energy [2].

Recently, MXenes have received paramount attention for their broad utility, e.g., optics and electronics, energy storage and conversion, environment and catalysis, biotechnology, and medicine [1]. MXenes exhibit biological properties associated with their carbon and/or nitrogen content, and the inertness of the transition metals has been called into question. Among the methods, a straightforward one to make MXenes more feasible, particularly for biotechnology, environmental, and energy-related applications, is to modify their composition and fabricate them through the use of green synthesis technology, as illustrated in Figure 1b [4]. Green synthesis is defined as a clean, safe, cost-effective, and environmentally friendly process of preparing micro- or nanostructured materials. Green synthesis is mainly used to minimize the toxic synthesis agents and employ an environmentally benign process [5,6,7]. Generally, microorganisms such as bacteria, yeast, fungi, algal species, and certain plants act as substrates for the green synthesis of nanomaterials [8]. In this route, the MXenes are non-toxic and have more excellent biocompatibility than the MXenes synthesized from conventional ways that use any hydrofluoric acid (HF)-containing HF-forming chemicals do. These HF chemicals may leak into groundwater resources and endanger aquatic life and humans by polluting the drinking water if the waste solutions are not adequately handled. Thus, studies examining simple, economical, and environmentally friendly ways to mitigate their potential toxicity are highly desired [9]. The green technology of MXenes is also predicted to be a cheaper, simpler, and energy-saving alternative to conventional chemical and physical methods, significantly impacting the realization of the MXenes’ uses [4,5,6].

Due to increasing research trends, green synthesis is now finding its pathway from the laboratory to commercial applications, but it still faces significant challenges. Thus far, no effective green synthesis protocol has been developed for MXenes. A more time-efficient, cost-competitive, and environmentally friendly fabrication of MXenes and MXene-based nanomaterials that allow the surface engineering of the nanostructures to be conducted for specific applications will be deemed useful. To this end, developing a viable green synthetic strategy can increase the MXenes’ versatility for various medical explorations related to treatment and diagnostic approaches, environmental applications, energy storage and conversion devices, and others. A viable strategy can be made by matching key specific properties to satisfy an appropriate utilization. The terminated groups influence the surface chemistry of the MXenes, i.e., F-functionalized MXenes are unsuitable for electrode materials in energy storage and catalysis systems because the F atom decreases their electrical conductivity. MXene synthesis requires modern protocols that are both efficient and feasible, including the use of a low-boiling solvent [10], low-energy processes through physical synthesis [11], low-temperature MAX preparation, and the possible utilization of biological substances, which were previously successfully used in the fabrication of other 2D materials such as graphene [12,13]. A recent fabrication and viable strategy for green MXenes can be achieved by manipulating the MXene’s diversity and green chemistry. These current green strategies include fabricating MXenes with milder etchants than HF for exfoliation, milder fluorine salts, and without fluoride [14,15]. Another approach is to use a hydrothermal method to synthesize large quantities of 2D MXene components without using toxic HF vapor, and combining Baeyer’s process with alkali-induced hydrothermal technology results in multi-layered Ti_3_C_2_T_x_ production [16]. Additionally, the MXenes can be synthesized via chemical vapor deposition and salt template or molten salt syntheses.

There are currently several critical reviews proposing the latest development of the synthesis of MXenes and MXene-based heterostructures for various applications, including properties and toxicity assessments [17,18,19,20]. However, despite this progress, only a few articles focus on the green and sustainable routes of MXene preparation. With an anticipated growing number of reports and the importance of the green technology of MXenes highlighting the non-toxic routes, low-temperature process, and energy-saving pathways, we summarize the recent advances of the green routes to prepare 2D MXenes through the low-temperature preparation of the MAX phase, HF-free MAX etching, electrochemical exfoliation, the direct synthesis of MXenes using a physical method, and potential biological substances. In addition, we propose and discuss emerging technology that utilizes green MXenes to emphasize the enhanced MXene performance obtained by green synthesis. Lastly, we provide the remaining challenges and future directions in developing the green MXene synthesis to stimulate advanced research.

## 2. Synthesis of MXenes

### 2.1. Conventional Synthesis of MXenes

**Figure 2:**
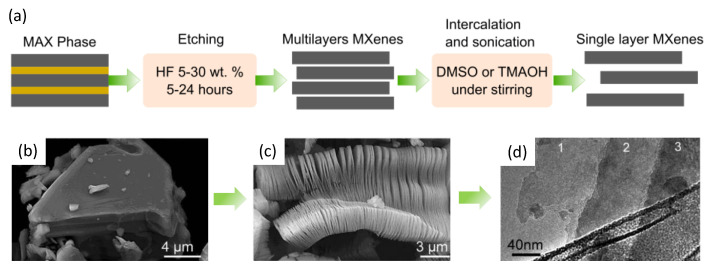
(**a**) General synthesis steps to produce single-layered MXenes. (**b**) Microstructure of MAX phase of Ti_3_AlC_2_ particle before treatment. (**c**) Ti_3_AlC_2_ after HF treatment and (**d**) Ti_3_C_2_ layers formed after HF treatment of Ti_3_AlC_2_ (a single sheet (monolayer) is the most transparent part of the sample). Adopted from reference with permission [22].

The lengthy process for the synthesis of MXenes begins with the preparation of bulk MAX phases as a starting material, as shown in Figure 2a. The MAX phases refer to layered polycrystalline of ternary carbides and nitrides with the general formula of M_n+1_AX_n_ (M = early transition metal, A = group III or IVA, and X = either carbon and/or nitrogen). They have an edge-sharing feature with a distorted M_6_X octahedra structure interlayered by group A elements. The MAX phases were discovered in the early 1960s [21], and they were successfully prepared with the formula of M_2_AC (Zr_2_TlC, Zr_2_PbC, Hf_2_TlC, and Hf_2_PbC), which was called the H phase. To date, there are more than 200 MAX possible compositions, having been mostly discovered by the use of theoretical calculations. However, only a few MAX phases have been successfully synthesized due to their thermodynamic instability. The microstructure of the MAX phase (Ti_3_AlC_2_) is visually shown in Figure 2b, and after HF etching (Figure 2c), it transforms into a Ti_3_C_2_ MXene nanosheet (Figure 2d). 

The M_n+1_AX_n_ (n = 1 and 2), Ti_2_AlC, and Ti_3_AlC_2_ are thermodynamically stable, and thus, they can be prepared in various temperatures. Meanwhile, in the Cr_n+1_AlC_n_ and Ti_n+1_SiC_n_ systems_,_ only Cr_2_AlC and Ti_3_SiC_2_ possess thermodynamical stability. The MAX phases are generally synthesized by solid-state reactions from their respective elements at high temperatures ranging from 1200 °C to 1800 °C, depending on their compositions. The solid-state reactions should be carried out for a minimum of 4 h in an inert atmosphere such as Ar or N_2_ gas. Metal powders such as Ti, V, Cr, Mo, etc., are typically used for the M source, but metal carbides such as TiC and Cr_3_C_2_ are becoming popular to reduce impurities [23,24]. It is also reported that the metal hydrides (e.g., ZrH_2_) are an alternative M source to synthesize Zr_2_AlC or Zr_3_AlC_2_, which seems very difficult using carbides [25]. The used metal hydrides should be performed with safety measures as the evaporated H_2_ may cause an explosion in large quantities. Al and Si are the most common A sources because of their low prices, although Ga and Ge are also used. The A elements should be added in excess of ~20 wt.% of their stoichiometric weight to compensate for the loss of thermal evaporation. As for the X elements, graphite is commonly utilized for carbide MAX with sub-stoichiometric quantities of 0.9%, and metal nitrides are the starting sources of the X elements for nitride MAX, but despite this, the nitridation of carbide MAX or mixing carbide and nitride MAX is also possible to conduct to form carbonitride MAX [26].

For the general etching process, hydrofluoric acid (HF) is the most common etchant to remove the A layer from the MAX phase. The exact etching condition depends on the type of MAX phase, HF concentration, temperature, and reaction time. Each gram of Ti_3_AlC_2_ MAX can be etched at room temperature to 40 °C for 24 h, 18 h, and 5 h by adding 5, 10, and 30 wt.% HF, respectively [27]. Unlike Ti_3_AlC_2_, the V_2_AlC MAX type should be etched at 60 °C in 40–50 wt.% HF for 96 h for each gram, which in some cases may induce defect formation [28], although it can recently be replaced with 12 mL of 48 wt.% HF and 8 mL of 12 M HCl within 72 h at 50 °C [27]. The hazardous HF concentration should be carefully handled as it can cause burns and damage organs and tissues. An effort to eliminate or at least reduce the use of HF can be achieved with a mixture of LiF and highly concentrated HCl. Still, treating the residual F waste remains challenging to prevent its release into water resources. After this process, the produced MXenes are still in a multilayer form with an accordion-like morphology. Expanding the interlayer spacing between the 2D MXenes with intercalating compounds is needed to delaminate the multilayer structures into single-layer MXenes flakes. Several compounds have been reported to be successful in the delamination of MXenes, typically dimethyl sulfoxide (DMSO), tetrabutylammonium hydroxide (TBAOH), and tetramethylammonium hydroxide (TMAOH). These final steps produce a colloidal solution containing electrostatically stable 2D MXene flakes, which can be solution processed for further use. The lengthy steps, energy-intensive processes, and careful handling that are needed to synthesize MAX and MXenes, as explained, have been massive drawbacks for the scalable production of MXenes. Thus, more eco-friendly and sustainable techniques for producing MXenes should be future requirements and interests when one is conducting research.

### 2.2. Synthetic Strategies towards MXenes with Different Dimensionality

**Figure 3:**
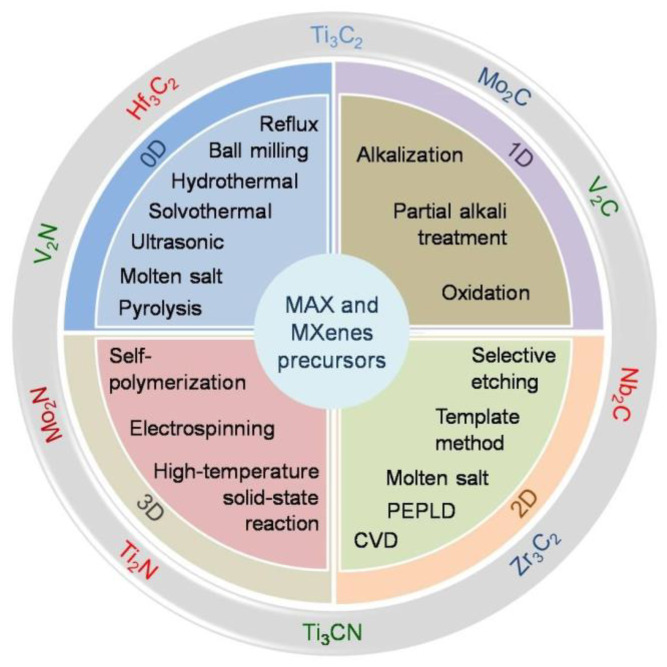
Compilation of recent fabrication methods of MXenes and derivatives depends upon their 0D, 1D, 2D, and 3D forms.

A comprehensive development list of MXene synthesis methods has been carried out to discover new types of MXenes and improve the functionality of the MXenes. The chemical and physical fabrication routes of MXenes could very much differ depending on the desired MXene compositions [1]. However, most of the synthesis methods of MXenes utilize a harmful chemical accessory that degenerates their functionality, notably for biomedical and environmental applications. Before discussing a more detailed strategy to obtain MXenes using green-related synthesis approaches, we provide an overview of the MXene synthesis methods attributed to their 0D, 1D, 2D, and 3D forms, as shown in Figure 3. The overview is related to the MXenes’ original, derivations, and nanocomposite forms.

In the quantum size regime, zero-dimensional (0D) materials are usually called quantum dots (QDs). The quantum size regime is attained when the dimensions of some of the materials are smaller than their exciton Bohr radius. MXenes are among the materials that can be transformed into QDs using various synthesizing methods, including top-down and bottom-up fabrication methods. The top-down approaches mean the use of physically or chemically bonded separation or breaking larger-sized materials into smaller ones. In contrast, the bottom-up processes imply the use of physically or chemically bonded arrangements or constructing the smaller (atomic/molecule) substances into a set of large-sized materials [29]. To our knowledge, only a few bottom-up methods have been successfully used to synthesize 0D MXenes, e.g., the molten salt synthesis method using molybdenum acetylacetonate, sucrose, and NaCl as parent precursors for the synthesis of the Mo_2_C QDs/carbon nanosheet composite [30]. Wang et al. successfully fabricated 0D MXenes using a bottom-up approach using a pyrolysis method [31]. They used molybdic acid, zinc acetate, and 2-methylimidazole as precursors to obtain Mo_2_C [31]. On the contrary, numerous top-down approaches have been conducted to obtain 0D MXenes, including liquid-phase exfoliation [32], hydrothermal [33,34], solvothermal [35], reflux or intercalation [36], ultrasonic [37,38], and ball milling methods [38,39] using 2D MXenes or directly using 3D MAX phases as the parent material. 

The one-dimensional (1D) material refers to the material that is crystallized in only one direction of the crystal growth. The size expansion of the 1D materials is negligible in two dimensions, but it is not restricted in other (third) directions. One-dimensional materials could be found with several characteristics, namely nanowires, nanoribbons, nanotubes, and nanorods. Even though MXenes are categorized into 2D materials, a recent study shows they could also be formed into 1D materials. Using an alkalization process, Lian et al. successfully transformed the Ti_3_C_2_ into 1D material [40]. They speculated that a continuous shaking treatment in an aqueous KOH solution could induce effective alkalization and delamination, essential in transforming Ti_3_C_2_ into a nanoribbon form [40]. Using a similar method, Dong et al. fabricated Ti_3_C_2_ MXene derivations in nanoribbons forms [41]. The two nanoribbons from Ti_3_C_2_ were M-NTO (or NaTiO_1.5_O_8.3_) and M-KTO (or K_2_Ti_4_O_9_), which were obtained using simultaneous oxidation and alkalization under hydrothermal conditions in NaOH and KOH solutions, respectively [41]. Nanocomposite-based 1D MXenes have also been previously reported. Using the partial alkali treatment of Ti_3_C_2_ in an NaOH solution, the MXene derivative of the Na_0.23_TiO_2_ nanobelt composed with Ti_3_C_2_ was favorably obtained [42]. Similarly, the alkali oxidation method successfully fabricated MXenes-derived TiO_2_ nanowires composed of Ti_3_C_2_ [43]. He et al. announced that they fabricated nanocomposites of a hydroxylated MXenes/carbon (h-Ti_3_C_2_/CNTs) nanotube using an alkalization process [44].

The two-dimensional (2D) materials are the materials that are not crystallized in only one dimension as they are not restricted to grow in the other two directions; the electron and hole motion is confined in only one spatial direction, whereas free propagation is allowed to occur in two spatial directions. It is clear that MXenes are 2D materials, and they were primarily found by the use of the top-down process using selective etching of the 3D MAX phase, as previously explained in many works of literature [1]. Nonetheless, bottom-up approaches also have been developed to obtain MXenes, e.g., the molten salt technique can realize more green MXenes as it can avoid any HF-containing HF-forming chemicals [1,45]. Compared to the top-down methods, the bottom-up methods are considered more suitable for synthesizing large-area 2D materials, they can be used to grow heterostructures of 2D materials directly, and they enable the growth of atomically thin non-layered materials. Various bottom-up methods of 2D materials have been developed, and it is possible to synthesize MXenes, such as by chemical vapor deposition (CVD), the template method, and plasma-enhanced pulsed laser deposition (PEPLD) [45].

Three-dimensional (3D) materials are materials that are crystallized in three directions of growth. MXenes as 2D materials could be transformed into the 3D form when they are highly stacked in the vertical direction. Druffel et al. synthesized 3D MXenes through a high-temperature solid-state reaction that enabled the construction of 3D crystals in high yield and purity with only fluoride ions terminating the layers [46]. Shang et al. [47] combined MXenes with rGO (graphene oxide) and facilitated the formation of a 3D structured hydrogel. As they are 2D materials, MXenes are challenging to construct in a 3D form, and it is even more difficult to do than it is for the other 2D materials because of the intrinsic properties of the MXenes [47]. Thus, MXenes are easier to transform into a 3D form when they are combined with other materials as a binder. Using electrospinning fabrication methods, Yuan et al. fabricated a flexible 3D MXene framework using a PVA/PEI mixture solution [48]. Zhang et al., on the other hand, successfully transformed a 2D T_3_C_2_T_x_ MXene into a 3D carbon-coated T_3_C_2_T_x_ architecture via the self-polymerization of dopamine over the surface of pristine 2D Ti_3_C_2_T_x_ which was achieved by a freeze-drying process and carbonization in an inert air atmosphere [49]. It was found that the self-polymerization of dopamine during the synthesis process enabled the transformation of 2D Ti_3_C_2_T_x_ into a 3D tremella-like form, and its subsequent carbonization could induce the perfect coverage of a thin carbon coating that protected the structure from air oxidation and structural aggregation [49].

## 3. Recent Progress in Green Synthesis of MXenes

### 3.1. Low-Temperature MAX Synthesis

The use of the MAX phase as the precursor for MXene is undeniable [50]. However, the conventional MAX phase synthesis is energy-intensive, requiring a temperature above 1400 °C with a minimum holding time of 4 h to allow a completed solid-state reaction to occur. Unless renewable electricity maintains the reaction temperature, the conventional MAX phase synthesis may emit a reduced concentration of CO_2_ into the atmosphere than when fossil-based electricity is used. Yet, in the long-term utilization of it, this unsustainable pathway will significantly contribute to air quality deterioration and global warming. Although the need for milder reaction temperatures for MAX phase synthesis is significant, it is difficult to obtain the high yields and purity of the MAX phase because the incomplete reaction of the precursor leads to the formation of secondary phases. Researchers recently have struggled to develop a techno- and energy-efficient approach to prepare MAX powder with a high yield and purity [50]. Herein, we discuss the recent progress of low-temperature MAX phase synthesis.

#### 3.1.1. Molten Salt Method for Green Production of MAX Phases

**Figure 4:**
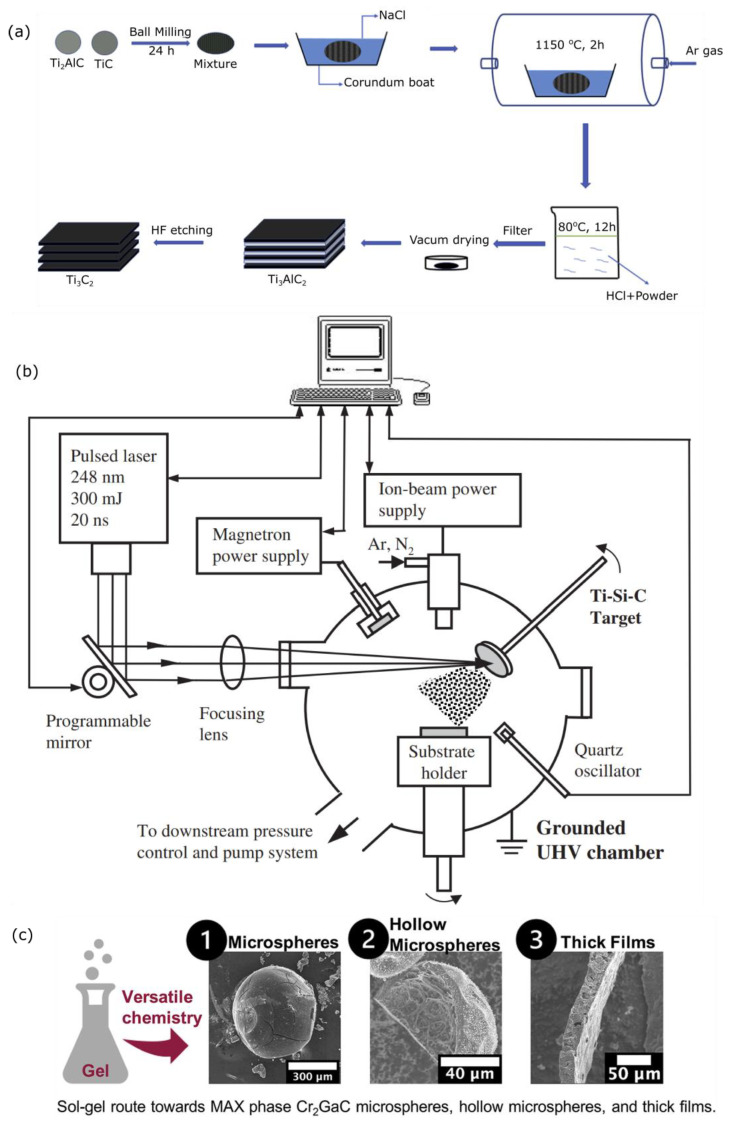
Low-temperature MAX synthesis; (**a**) molten salt method with lower synthesis temperature of MAX-phase Ti_3_AlC_2_ by 200 °C [55], (**b**) experiment setup of the magnetron sputtering assisted pulsed laser deposition (PLD) system [67], and (**c**) the result from MAX phases synthesized by using biopolymer sol-gel synthesis [74].

Molten salt is an emerging alternative for preparing a wide range of non-oxide powders [51]. It is a modification of powder metallurgy where low melting point salts are added to the reactants. The liquid salt, when it melts, provides an effective medium to facilitate the homogeneous dissolution and diffusion of the reactants, yielding a lower reaction temperature with high purity [52,53]. The molten salt method also eliminates the use of ball milling pre-treatment. Some molten salts that have been widely utilized include metal chlorides, metal carbonates, metal nitrates, and metal fluorides. Two different Ti-based MAX powders (Ti_2_AlC and Ti_3_AlC_2_) were successfully prepared at 950 °C for 5 h from Ti, Al, and acetylene black as raw materials in an NaCl–KCl system as a salt medium with the reaction time that can be shortened to 2 h if the reaction temperature is raised to 1000 °C [54]. Ninety-six point seven percent purity of the Ti_2_AlC and Ti_3_AlC_2_ powders was achieved [53]. Liu and co-workers increased the purity of Ti_3_AlC_2_ by changing the reactant mixture to Ti_2_AlC–TiC in a molten NaCl-assisted synthesis, but the reaction temperature should be increased to 1150 °C as shown in Figure 4a [55]. Recently, Ti_3_AlC_2_ MAX powders with high purity of 98.5% were successfully produced by the microwave-assisted molten salt method (MA-MS) using a TiH_2_/Al/1.8TiC mixture as the reactant materials and NaCl–KCl as the molten salt at 1050 °C [52]. The microwave assistance enables the formation of Ti_3_AlC_2_ powder in a relatively short reaction time (30 min), thus breaking the record for the fastest Ti_3_AlC_2_ powder preparation so far at this temperature. In this method, the Ti_3_AlC_2_ formation is significantly influenced by the heating temperature and mass ratio of the raw materials to molten salts, where a higher temperature and a higher proportion of molten salts are preferable to producing a homogeneous, high purity, and high yield of Ti_3_AlC_2_ powder [54]. The molten salts-assisted Ti_3_AlC_2_ synthesis can be extended to prepare other MAX elements. For instance, Ti_3_SiC_2_ powders that are usually conventionally prepared at 1350 °C were instead prepared at 1200 °C with the assistance of an NaCl flux [56]. A similar reaction temperature reduction has been achieved for Ti_2_AlN, V_2_AlC, V_2_SnC, and Cr_2_AlC syntheses [57,58,59,60]. This method can be predictably expanded to many more MAX families, and it could be a standard for industrial production because of its eco-friendliness, sustainability, and energy-effective features. 

#### 3.1.2. Physical Method for Rapid Production of MAX Phases

The physical methods offer a more robust, rapid, and lower reaction temperature to produce the MAX phase, either in bulk or thin film forms. However, the physical methods for the growth of the bulk and the thin film of the MAX phase are still limited, yet few studies have shown the potentiality for an industrially relevant process. In the thin film form, physical vapor deposition (PVD) becomes an alternative for the moderate temperature (700–1000 °C) synthesis of MAX even though some group 5 or group 6 M elements such as V_2_GeC, Cr_2_GeC, and Cr_2_AlC can be physically deposited at 500 °C [61,62,63]. Some reports show that the bulk Ti_3_AlC_2_ and Cr_2_AlC MAX phases have been well prepared by spark plasma sintering (SPS), pulse discharge sintering (PDS), and microwave methods with the reaction temperature ranging from 1050 °C to 1300 °C for up to 60 min [64]. Even with the microwave hybrid methods, the ultrafine and high-purity Cr_2_AlC powders were synthesized at 1050 °C for 3 min, suggesting that this is a promising industrial-scale production system [23]. The rapid synthesis of Ti_3_AlC_2_ powder was demonstrated by the pulse discharge sintering of the Ti/Al/C powder mixture at a molar ratio of 3:1.1:1.8, performed at 1250–1350 °C for 15 min and 1300 °C for 15–60 min [65]. However, some intermediate phases, such as AlTi_3_, AlTi, Ti_3_AlC, and Ti_2_AlC, can be observed. The rapid synthesis still has the remaining drawback of having impurities that can influence the overall performance of the exfoliated MXenes. In a thin film form, some efforts have been carried out to reduce the reaction temperatures to 400–600 °C to reduce the oxidation of specific substrates such as stainless steel. To this end, magnetron sputtering is considered a facile processing method for thin film deposition due to its easy process and having tunable control of the phase purity and composition [50]. In this method, the M and A sources are typically in a metal form, and the X source is typically graphite to achieve flexible control of the MAX phase composition. It is likely applicable at the industrial scale due to its simplicity and robustness. For nitride MAX, the magnetron sputtering uses N_2_ gas as an N source, and it eliminates the use of graphite. Combining the use of N_2_ gas and graphite can produce carbonitride MAX. The polycrystalline V_2_AlC phase was obtained by this method with a nearly stoichiometric composition [66]. Interestingly, magnetron sputtering could be combined with pulsed laser deposition (PLD), as shown in Figure 4b [67]. In this hybrid system, the PLD could potentially be used for MAX production below 300 °C due to the high-power pulsed laser beam that evaporates the MAX phases; thus, this subsequently deposits the MAX vapor on the substrate [67]. Besides magnetron sputtering, CVD was actually the earliest method that was used to prepare thin film MAX when Nickl et al. successfully prepared Ti_3_SiC_2_ [68], which was then conducted by other researchers [69,70]. However, the CVD process requires much higher temperatures (1000–1300 °C) to produce thin film MAX than that of magnetron sputtering, and this has become a challenge that we need to tackle despite its excellent control of the crystal thickness, and this has been underlined by the recent 2D ultrathin Mo_2_C synthesis [71].

#### 3.1.3. Sol-Gel-Based Synthesis for Nanoparticulate MAX Phase

Sol-gel chemistry has been widely utilized to synthesize various solid-state materials for multifunctional applications. It alternatively replaces the conventional solid-state reactions for its controllability of homogeneous and nanosized particulate production. The method offers the requirements of a lower temperature and a shorter reaction time as the reaction starts at the atomic/molecular level, allowing a more straightforward diffusion path and faster mass transport to occur [72]. Sol-gel may be an option for low-temperature MAX synthesis. The bulk Cr_2_GaC MAX phase is usually produced at a high purity at 1000 °C for multiple, consecutive days, which is energy and time-consuming. The sol-gel method otherwise synthesized the Cr_2_GaC MAX phase, which was followed by calcination at 600–900 °C for 5 h. The obtained morphology using the sol-gel process differs from those MAX phases of the conventional high-temperature solid-state reaction. The sol-gel method can observe the needle-like particles (length = 1600 nm and width = 200 nm) with a typical layer structure. The enhanced solution-processable precursor mixture is an interesting feature of sol-gel-synthesized Cr_2_GaC MAX. This milder synthesis process can also be applied to prepare Mn-containing solid solutions of the MAX phase Cr_2_GaC ((Cr_1−x_Mn_x_)_2_GaC) with Mn amounts ranging from 2 to 20 wt.% in the M layers [73]. However, with Mn dopant, there are different impurity phases, such as Mn_23_C_6_, CrO_2_, MnO, and CrC, that may be removed by further acidic treatments. The morphology of Cr_2_GaC MAX can be turned into microspheres, hollow microspheres, and thick films by adding biopolymer templates (e.g., chitosan dan carboxymethyl-dextran) that add the surface feature of the sol-gel-assisted Cr_2_GaC MAX synthesis, as shown in Figure 4c [74]. Two more MAX phase members, V_2_GeC and Cr_2_GeC, have been favorably synthesized with high purity and yield [75]. The expansion of the sol-gel method in preparation for the MAX phase will be a crucial step toward its applicability.

### 3.2. Replacing HF Etchant with Safer Chemicals

**Figure 5:**
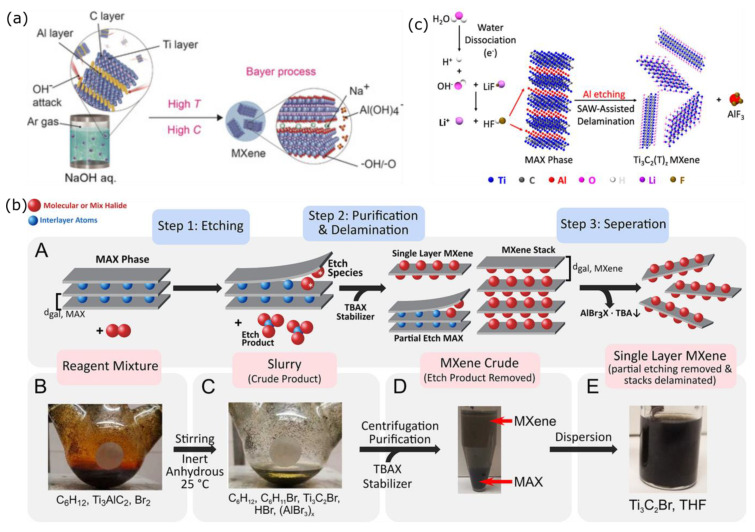
(**a**) According to the Bayer method, high temperatures synthesis and high NaOH concentrations support the dissolution of the Al (oxide) hydroxides in NaOH [76]. (**b**) The process for the formation of delaminated halogen-terminated MXenes; (A) adding Br_2_ to Ti_3_AlC_2_ in anhydrous cyclohexane generates a deep red solution; (B) Br_2_ reacts with the Al interlayer inducing the supernatant thus turns to a pale yellow color, indicating a depletion of Br_2_ and the construction of AlBr_3_; (C) AlBr_3_ is rendered inert by addition of stabilizers (tetrabutylammonium bromide, TBAX); (D) the MXene crude is purified by repeated redispersion in non-polar solvent (i.e., CHCl_3_); (E) the purified size-selected MXene is obtained by centrifugation and dispersion process in THF [82]. (**c**) The evanescent electric field and surface acoustic waves (SAW) field induce dissociation of the water molecules comprising the MAX phase to generate hydroxyl free radicals and protonated species. The presence of LiF can induce the localized “in situ HF” that selectively etches away the Al in the Ti_3_AlC_2_ MAX phase. This delamination process occurs under strong mechanical vibration together with the SAW, which accelerates the substrate surface in the order of about 10^8^ ms^−2^ [83].

Earlier attempts to synthesize MXenes involved toxic chemicals such as HF, which penetrated the skin and tissue. The liquid phase exfoliation of 2D materials also could be performed by using low-boiling-point solvents that are environmentally friendly, e.g., water, ethanol, and isopropanol [10]. Non-HF etching alternatives, such as a high-temperature NaOH etching technique, as shown in Figure 5a, indicate a high temperatures synthesis and high NaOH solvent concentrations that will support the dissolution of the Al (oxide) hydroxides in NaOH [76]. Electrochemical hydrochloric acid (HCl), hydrothermal HCl, and molten-salt ZnCl etchings were also recently proposed for the green production of MXenes [76,77]. The hydrothermal etching technique has been employed to synthesize Ti_3_C_2_ MXenes using non-toxic etching agents (NaBF_4_, HCl), enabling efficient and safe MXene exfoliation and widespread 2D MXene use [78]. Guo and their team conducted MXene etching in hydrothermal conditions and utilized F-based etching solutions in combination with HCl [79]. Li and coworkers combined MXene synthesis and a battery fabrication protocol through the one-step process of exfoliation inside a battery using an F-rich electrolyte to avoid it being a non-eco-friendly and multistep process [80]. Feng et al. prepared, in situ, nitrogen-doped Ti_3_C_2_ MXene QDs via amine-assisted solvothermal tailoring. Exfoliating pure MXenes results in the formation of MXenes with layers. Apart from the green approach, this may guide the large-scale fabrication of fluorescent MXene QDs doped with elements of interest [35]. Pang and coworkers investigated MXene applications via a non-toxic, HF-free synthesis protocol, namely, thermal electrochemical etching, to synthesize universal MXenes (e.g., Ti_2_CT_x_, Cr_2_CT_x_, and V_2_CT_x_) [81]. The prepared MXenes demonstrated functionality suitable for an aqueous rechargeable battery.

The molten salt system is considered a safer and more efficient method of preparing MXenes. Extending the range of the parent MAX phases creates additional tuning space for the MXene’s functionalities and properties. It enables the synthesis of novel MXenes that are difficult to obtain via conventional methods [84]. Another group of researchers produced water-dispersible 2D MXene nanosheets with molten salt etching utilizing SnF_2_ [85]. A recent report shows the ability of halogen in anhydrous media to synthesize MXenes from the MAX phase at room temperature, as shown in Figure 5b [82]. Halogens (Br_2_, I_2_, ICl, and IBr) in anhydrous media could etch Ti_3_AlC_2_ into MXenes via a radical-mediated process that depends on the molar ratio, the absolute concentration of the halogen and solvent, and the temperature. The etching method using halogens opens up opportunities to obtain more green MXenes than the HF-containing media can [82]. In addition, Ghazaly and their team successfully established a one-step synthesis with rapid local Ti_3_AlC_2_ MAX phase-to-Ti3C2Tx MXene conversion, which was aided by proton generation via solution dissociation in the presence of megahertz acoustic excitation (see Figure 5c) [83]. Acoustic forcing aids in the selective etching of the MAX phase into MXenes [83]. The MXenes’ potential utility is contingent upon developing more efficient synthesis procedures. Likewise, microwave-assisted solution synthesis also can facilitate green substance solutions to synthesize MXenes with a faster synthesis process. 

### 3.3. Electrochemical Exfoliation for HF-Free Etching Process of MXenes

**Figure 6:**
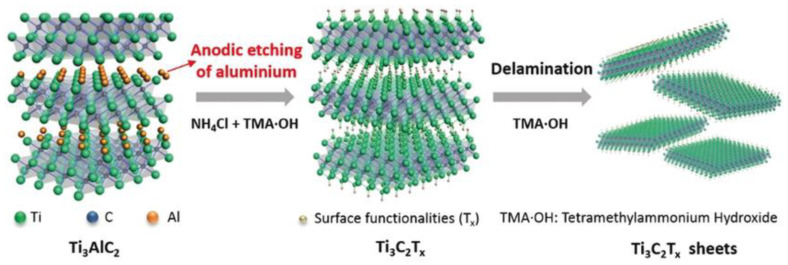
Electrochemical exfoliation for HF-free etching process of MXenes [86].

The A layer selective etching and exfoliation process of the bulk MAX phase without involving an HF etchant has been introduced by Yang et al., as shown in Figure 6 [86]. They used the anodic corrosion concept for an F-free electrochemical etching method for Ti_3_AlC_2_. In the binary aqueous electrolytes system, Ti_3_AlC_2_ was successfully and efficiently exfoliated into 2D Ti_3_C_2_T_x_ (T = O, OH). Here, 1 M ammonium chloride (NH_4_Cl) and 0.2 M tetramethylammonium hydroxide (TMAOH) electrolytes were used as the mixtures. The presence of chloride ions is essential to break the Ti-Al bonds by etching the Al layer under anodic conditions, as shown in the following reaction: Ti_3_AlC_2_ − 3e^−^ + 3 Cl^−^ = Ti_3_C_2_ + AlCl_3_. Subsequently, the OH^−^ species from ammonium hydroxide (NH_4_OH) produced in situ intercalated the interlayer space, yielding 90% of the exfoliated 2D Ti_3_C_2_T_x_ (T = O, OH) flakes with a lateral size of over 2 μm and a thickness of 1.3 nm. This size exceeds the exfoliated flakes obtained by conventional HF-etching. Ti_3_AlC_2_ can also be electrochemically etched in room temperature ionic liquid (RTIL) electrolytes consisting of [BMIM][PF_6_] and MeCN [87]. Compared to conventional etching, which requires more than 24 h to complete the Al removal, the ionic liquid electrochemical etching shortens the reaction time to 5 h with comparable Ti_3_C_2_T_x_ flakes (with a lateral size of 820 nm). The produced Ti_3_C_2_T_x_ flakes exhibited good electrochemical performance, indicating the reliable practicability of this green and mild synthesis method of MXenes. Following this report, V_2_AlC MAX was etched in a closed coin-type CR2030 cell with a mixture of 21 M LiTFSI + 1 M Zn(OTf)_2_ as the electrolyte and Zn metal as the anode [80]. With 400 cycles of charging/discharging at 10 A g^−1^, V_2_AlC MAX was electrochemically converted in situ into V_2_CT_x_ flakes with lateral sizes ranging from 1 to 5 µm and a thickness of 8.5 nm. The Al layer was attacked by F^−1,^ which was derived from OTf^−1^, thereby breaking the V–Al bond through the following reaction formula: V_2_AlC + y F^−1^ + (2x + z)H_2_O − (y+3)e^−^ → V_2_C(OH)_2x_F_y_O_z_ + Al^3+^ (Al_2_O_3_, AlF_3_) [80].

The HF-free etching via electrochemical exfoliation was also used to prepare green Nb_2_CT*_x_.* First, the bulk Nb_2_AlC MAX phase was deposited onto carbon fiber cloths (CFCs). With 1 V anodic potential, the Al layer was selectively etched by 0.5 M HCl electrolyte at 50 °C according to the following: Nb_2_AlC + yCl^−^ + (2x + z) H_2_O → Nb_2_C(OH)_2x_Cl_y_O_z_ + Al^3+^ + (x + z) H_2_ ↑ + (y + 3) e^−^ [88]. Compared with the conventional HF etching method, the electrochemically etched method offered a higher degree of Al layer removal, evidenced by the vanished Al *2p* XPS peak [88]. The F-free Nb_2_CT_x_ brings advantages to its application as a biosensor, e.g., it has enhanced biosensor stability and reproducibility. As surface terminations impact the MXenes’ properties, the modification of the surface functional groups beyond –OH, and –O may disclose the novel physicochemical features of the MXenes. As previously discussed, molten salts are promising options that can be used to modify the surface properties of the MXenes obtained from electrochemical etching. When the inorganic salts of AlCl_3_ are used, the chlorine ions (Cl^−^) effectively oxidize the Al layer via Ti_3_AlC_2_ +3Cl^−^ → AlCl_3_ ↑ + Ti_3_C_2_ + 3 e^−^ [89]. The Cl then governs the surface termination of Ti_3_C_2_ into Ti_3_C_2_Cl_2_. The surface terminations can be in situ modified from –Cl to –O and/or –S by adding Li_2_O and/or Li_2_S, respectively, which shortens the modification routes and adds a greater variety of surface functional groups of other MXene families. 

### 3.4. Direct Synthesis of MXenes by Physical Methods

Instead of wet synthesis, dry etching methods may become a more promising solution to obtain MXenes as they minimize the use of chemical solvents. The bottom-up and/or top-down fabrications using physical methods may also work, i.e., the molten-salt and pyrolysis methods can fabricate the 0D form of MXenes, and in comparison, CVD and PEPLD could likely be used to directly grow 2D-type materials with large areas, and it is easy to synthesize heterostructures-based 2D materials and enable them to grow atomically thin-layered 2D materials [45]. Most of the physical synthesis methods, including ball milling, melt mixing, PLD, MBE, sputtering, etc., are less toxic than the solutions-based fabrication methods are, which means that they could possibly be utilized in biomedical and environmental-related applications. For instance, 2D MXenes thin film grows using vacuum methods, and they can work as biosensor elements for biomarker diagnosis applications [90]. 

**Figure 7:**
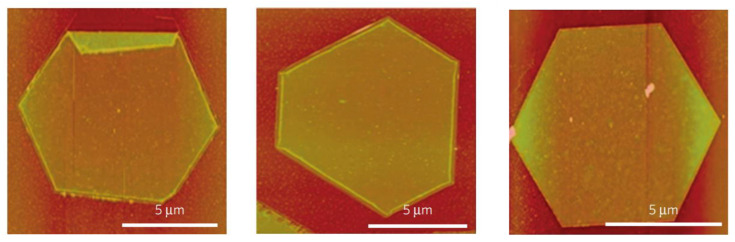
AFM images of the hexagonal ultrathin α-Mo2C crystals are synthesized using direct synthesis via the CVD method using carbon source from methane and a layered Cu/Mo foil as the substrate [71].

The bottom-up construction of 2D ultrathin α-Mo_2_C MXenes with large areas via the CVD method using a carbon source from methane and a layered Cu/Mo foil as the substrate has been previously reported, as shown in Figure 7 [71]. Magnetron sputtering is also used to grow Mo_2_C thin film directly using the Mo_2_C target [91]. Ti_3_C_2_ MXenes thin films, on the other hand, have been successfully fabricated by etching the Ti_3_AlC_2_ thin films, which were previously grown using three elemental targets (Ti, Al, and C), utilizing DC magnetron sputtering [92]. Some types of MXene thin films have also been successfully fabricated using other thin film deposition methods, such as the MAX phase of Ti_3_AlC_2_ thin films deposited from three Ti, Al, and C targets using DC magnetron sputtering, and then, they were etched into the Ti_3_C_2_ MXene thin film [92]. Mo_2_C thin film can also be grown on a quartz plate substrate via magnetron sputtering directly using the Mo_2_C target [91]. Other types of sputtering methods, such as ion beam sputtering (IBS), have been used to fabricate MXenes. IBS uses an ion source (Ar^+^) for the generation of sputtered particles of the MXenes targets, while the growth of the thin films on the substrates is spatially separated [93]. The PVD type of fabrication method used to fabricate MXenes is PLD. Recently, single-crystalline Mo_2_C thin films on the sapphire substrate have been epitaxially grown using one type of PLD technique, a plasma-enhanced PLD (PEPLD), while an Mo metal was used as the target [94,95]. In this case, by equipping the PLD system with a high-voltage electrode at the methane inlet of the chamber, the PLD system can ionize the gas when it is vented into the chamber, and thus it can produce plasma. The atomic layer deposition method (ALD) has also been reported experimentally and theoretically to grow high-quality 2D carbide materials, including MXenes and MXene derivatives [96,97,98,99]. ALD is a suitable technique for fabricating scalable electronic devices since it enables the synthesis of an atomic scale of thin film thickness and accuracy control of the thin film composition and allows us to produce a thin film with the perfect step coverage [97].

### 3.5. The Promise of the Bioagents as Exfoliation, Reduction, Capping, and Stabilizer Agents

**Figure 8:**
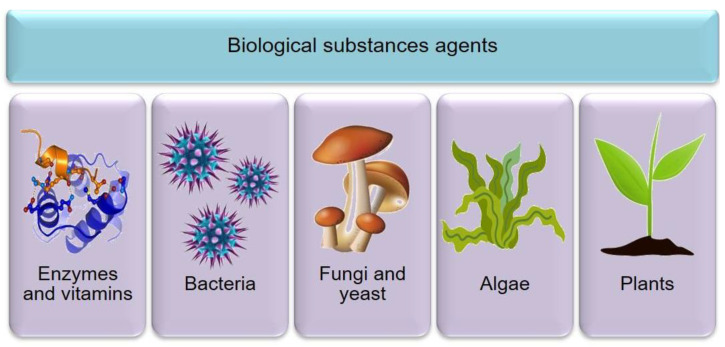
Green synthesis of low dimensional materials via enzymes, vitamins, bacteria, fungi and yeast, algae, and plants as the possible reduction, capping, stabilizer, and exfoliation agents.

Biological substances could be used as primary tools or agents to synthesize many kinds of low-dimensional materials such as QDs, 1D, and 2D materials. In this case, biological substances would potentially be the etching or reducing agents to stabilize the low-dimensional materials. Several previously reported biological substances could be used to synthesize low-dimensional materials: enzymes, vitamins, bacteria, yeasts, fungi, algae, and plants, as summarized in Figure 8. Enzymes have been widely used to synthesize nanoparticles (NPs) such as Ag, Au, and Fe/Pd nanocomposites [5]. Moreover, green tea extracts have been utilized lately as reductive and capping agents to obtain a bimetallic Fe/Pd NPs composite [100]. Vitamins are one of the biological substances that can be used as a stabilizing agent to synthesize nanoparticles. Vitamin B2 has been found recently to be capable of acting as the reducing agent for synthesizing nanowires and nanorods [5]. Ascorbic acid-chitosan can serve as a capping, reducing, and stabilizing agent to synthesize Ag NPs [101]. Living biological substances such as bacteria and actinomycetes are recognized as agents that are used to synthesize low-dimensional materials, especially NPs [4,5]. A variety of bacteria can be used to synthesize metal NPs, such as Lactobacillus casei, Bacillus cereus, E. coli, Bacillus subtilis, Magnetospirillum magneto tacticum, Aquaspirillum magnetotacti cum, Aspergillus favus, etc. [4]. Other living biological substances that can be used to synthesize low-dimensional are yeasts and fungi. Fungi enable the production of more significant amounts of NPs compared to bacteria [4]. Numerous fungi are capable of synthesizing NPs, such as ZnO NPs using Aspergillus strain, Candida albicans, and Aspergillus terreus, Ag NPs are synthesized using Trichoderma viride, Fusarium oxysporum, and Arthroderma fulvum, while TiO_2_ NPs may be synthesized using Aspergillus flavus [4]. Algae have also been reported as stabilization agents of metal NPs, such as S. platensis protein which is used to synthesize Au NPs [102]. Plants are also known as stabilization agents, which are used to synthesize NPs through their phytochemicals [6].

Undeniably, in most cases, the 2D forms of MXenes can be achieved using wet or solution chemical etching. Even though the former results that have been explained are more related to metallic NPs, numerous 2D materials have also been successfully synthesized using green synthesis methods. For instance, Chufa et al. have successfully fabricated graphene oxide using the methanol-extracted Vernonia amygdaline plant leaf [103]. Chen et al. used chlorophyll extract from Sapium sebirefum leaves as an exfoliation agent to synthesize 2D materials graphene, MoS_2_, and h-BN [104]. No reported etching media from biological substances are utilized these days to synthesize MXenes. Nevertheless, we believe several biological substances may be used to fabricate MXenes as etching media in the future.

## 4. Potential of Green MXenes for Environmental, Biomedical, Electronics, and Energy-Related Applications

To date, the green-related synthesis of MXenes-based materials is mostly achieved by replacing the HF etching agent with less toxic etching agents, including NaOH, NaCl, ZnCl, NaBF_4_, LiF+HCl, etc. [76,77,78,79], or with a chemical-free etching agent, including thermal electrochemical etching [81]. Those green synthesis approaches have several advantages, such as less toxicity, i.e., the use of non-toxic etching agents (i.e., NaBF_4_+HCl) and avoiding the usage of strong acid HF significantly decreases the toxicity of the chemical, and it is an environmentally friendly, facile synthesis due to the use of non-toxic etching agents, and the experimental process is relatively easy when it is compared to use of the toxic etching agent. In the usage of toxic etching agents, special attention during the practical to prevent the explosion should be taken. The use of non-toxic agents has a low cost, i.e., the usage of non-toxic etching agent eliminates pieces of the equipment, making it more cost-effective; commercialization is an important factor, i.e., using a non-toxic etching agent, which is less toxic, easy to handle, and has a low cost, realizing the green synthesis of MXenes-based materials potential for commercialization. Here, we discuss the potential applications and the demand for green MXenes in some applications below.

### 4.1. Application of the Green MXenes for Environmental Remediation and Catalysis

**Figure 9:**
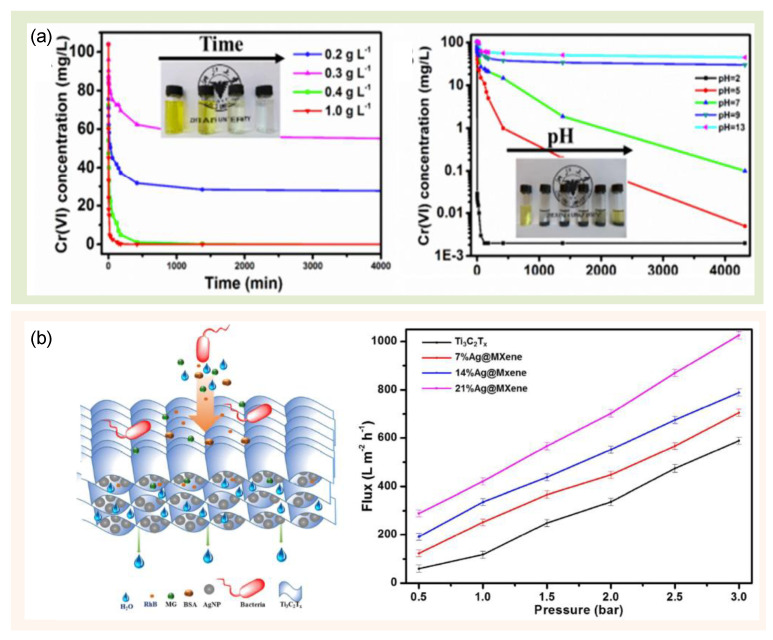
The applications of MXenes in environmental remediation. (**a**) Dosage effect of the Ti_3_C_2_T*_x_*−10% nanosheets on removing Cr (VI) in 400 mL of 104 mg L^−1^ solution (pH 5.0) (left-hand side panel) and the insert panel is the corresponding photograph with elongation of time, indicating the change in color in the case of 0.4 g L^−1^ Ti_3_C_2_T*_x_*−10% nanosheets. Removal of Cr (VI) in 400 mL of 104 mg L^−1^ solution (Ti_3_C_2_T*_x_*−10% nanosheets concentration: 0.4 g L^−1^) dependent on pH (right-hand side panel), and the insert is the final solution color at different pH [107]. (**b**) MXenes/silver nanoparticles (AgNPs) composite for water permeation with anti-fouling properties is schematically shown on the left-hand side panel [109]. The pure water flux of Ti_3_C_2_T_x_ and MXenes/AgNPs composite membranes is shown in the right-hand side panel [109].

Environmental remediation is one application products of green technology. MXene is a novel material that has a lot of potential for environmental remediation purposes. Thus, both the developments of green synthesis and green technological aspects of MXenes are necessary to discuss in this review article. The layered structure and composition of MXenes generate distinctive physical properties such as hydrophilicity, excellent electronic and ionic conductivity, remarkable flexibility and mechanical stability, and a highly accessible surface area [105]. Yet, their interlayer spacing is tunable. The presence of sites for direct ion exchange and selectivity towards specific pollutants [105,106] offers inimitable benefits as water treatment materials [105]. MXene-based adsorbents are applicable for eliminating noxious organic pollutants, heavy metals, and radionuclides [106,107]. MXenes are possibly regenerated by a simple acid or base treatment, which opens the possibility of reusing them in manifold cycles with a high maintenance capacity [106]. Many results have been reported on MXene-based adsorbents, which provide an excellent possibility for them to be used as water treatment materials, and we present an example in Figure 9a about Ti_3_C_2_T_x_ nanosheets removing Cr (VI) [107]. Another example is the MXene/PVDF membrane, which is utilized for environmental applications in the charge- and size-selective rejection of ions and molecules [108]. This material excellently selects single-, double- and triple-charged metal cations and different dye cations [108]. Moreover, the MXene/silver nanoparticles (AgNPs) composite is also developed for water permeation as it has anti-fouling properties [109]. As shown in Figure 9b, the DI water was filtered at differential pressures in the range of 0.5 to 3 bar, indicating a linear dependence on the pressure and high structural stability of the pore channels [109]. It also shows that the pure water flux for the 21% of the MXene/AgNPs composite membrane was ~3.55 higher compared to the pristine MXene membrane at 1 bar of pressure [109]. AgNPs are reported to act as a slit interspace between the MXene nanosheets to afford extra nanopores for water permeation, and thus, they increase the water flux to about ~420 L m^−2^ h^−1^ bar^−1^ as well as enhance the rejection of methyl green up to 92.32% and Rhodamine B at about 79.93%. Many MXene-based adsorbents are discussed in previous original papers and reviews [105].

The antibacterial activity of MXenes, especially in the form of a membrane, could endorse their potential application as the anti-biofouling membrane in water treatment processes [110]. In a previous result comparison with PVDF, the antibacterial rate of a fresh Ti_3_C_2_T_x_ MXene is reported to be up to 73% against B. subtilis and 67% against E. coli, while aged Ti_3_C_2_T_x_ MXenes obtain about 99% of the growth inhibition of both of the bacteria [110]. The brief explanation above shows that the lowering or removal of the toxicity in the MXenes is the key to realizing MXenes for environmental remediation. The potential toxicity of MXenes is mainly from the chemical synthesis production, which may contaminate the MXenes. In this matter, the green synthesis approach is very important to remove the toxic elements contained in MXenes, and the safety of MXenes as the tools for environmental remediation could be, therefore, achieved.

### 4.2. Application of the Green MXenes for Biomedical, In Vitro, and In Vivo Studies

**Figure 10:**
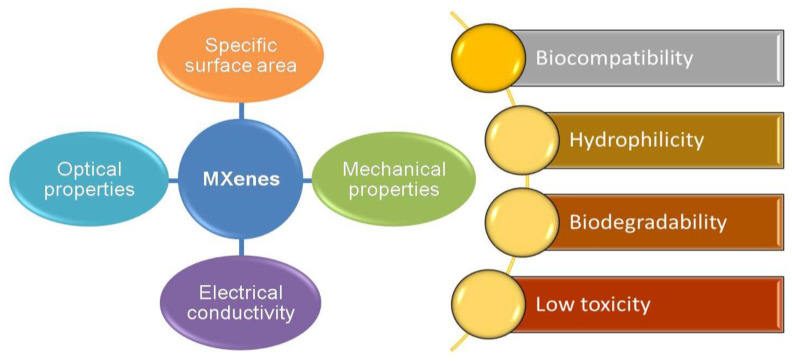
Properties of MXenes and MXene-based materials for biotechnology-related applications.

One implementation of the green synthesis and green technological aspects of MXenes could be found in their applications for biomedical purposes. However, similar to that of most other 2D materials-based biotechnology and biomedical applications, the real clinical applications of MXenes are still lacking, and they are limited by the empty space in the knowledge of integrating MXenes with living biological systems, blurred biological mechanisms, and their potential toxicity [33]. Nevertheless, the MXene is a new material class with significant potential applications in healthcare, biomedical engineering, and tissue regeneration [2]. MXenes show favorable biological properties, including a large surface area for drug loading/delivery, water-soluble biocompatibility, and additional electronic properties for CT and MRI scans. The appealing physicochemical and biocompatibility properties of the novel 2D materials have rekindled interest in biomedicine and biotechnology research. Figure 10 compiles the unique properties of MXenes and MXene-based materials, which can support their use in biotechnology applications. MXenes can serve in vivo and in vitro biomedical applications such as drug delivery, heat delivery, contrast agents for cancer treatment, and sensors for health monitoring. Though not so many previous reports use the green technology approach in realizing MXenes for biomedical applications, several attempts have previously reported the application of MXenes in the biomedical fields. For example, Ti_3_C_2_T_x_ MXene quantum dots (MQD) could be naturally delivered into a human vascular endothelial cell within 24 h of the cell culture. The localization of the Ti_3_C_2_T_x_ MQD is highly stable and promising for nanomedicine applications, such as drug delivery [33]. Ti_3_C_2_ MXenes have a high drug-loading capability of up to 211.8% and exhibit pH-responsiveness and a near-infrared laser-triggered on-demand ability for drug release. Ti_3_C_2_ MXenes have a high photothermal conversion capability, and they are applicable for efficient tumor eradication via synergistic photothermal ablation and chemotherapy [111]. Ti_3_C_2_ MXenes can act as a contrast agent to detect a tumor spot in real-time via bioimaging during cancer treatments. Thus, the combinatory treatment of photothermal therapy, chemotherapy, and real-time bioimaging using MXenes could significantly enhance the effectiveness of cancer treatments [112]. MXenes also have an anti-bacterial ability against some Gram-positive and Gram-negative bacteria, which is becoming an essential feature of MXenes for biomedical applications [113].

By adjusting the fabrication and optimizing parameters, the properties of MXenes can be modified for precise applications, i.e., biosensors that are also applicable in medical applications [114]. On the subject of biosensors, a recent finding reported that an MXene/platinum nanoparticle (PtNPs) nanocomposite thin film deposited on GCE electrode substrate (Ti_3_C_2_T_x_/PtNPs/GCE) exhibits excellent electro-catalytic activity and shows significant sensitivity to various biological and organic substances, such as dopamine, ascorbic acid, uric acid, acetaminophen, and H_2_O_2_. The features of having great electro-catalytic and sensitivity could be far superior when they are used for biosensors and biofuel cell applications [115]. Moreover, a study also reported an amperometric biosensor based on an MXenes-acetylcholinesterase (AChE)-chitosan (CS) nanocomposite-modified glassy carbon electrode (AChE/CS/Ti_3_C_2_T_x_/GCE) could be used for the detection of an organophosphorus pesticide—malathion [116]. Other than that, MXenes are sensitive to detecting volatile organic compound (VOCs) gas, further revealing their breath-based biomarker diagnosis applications [90]. Indeed, in most cases, MXenes could be used for many applications when they are combined with various materials.

**Figure 11:**
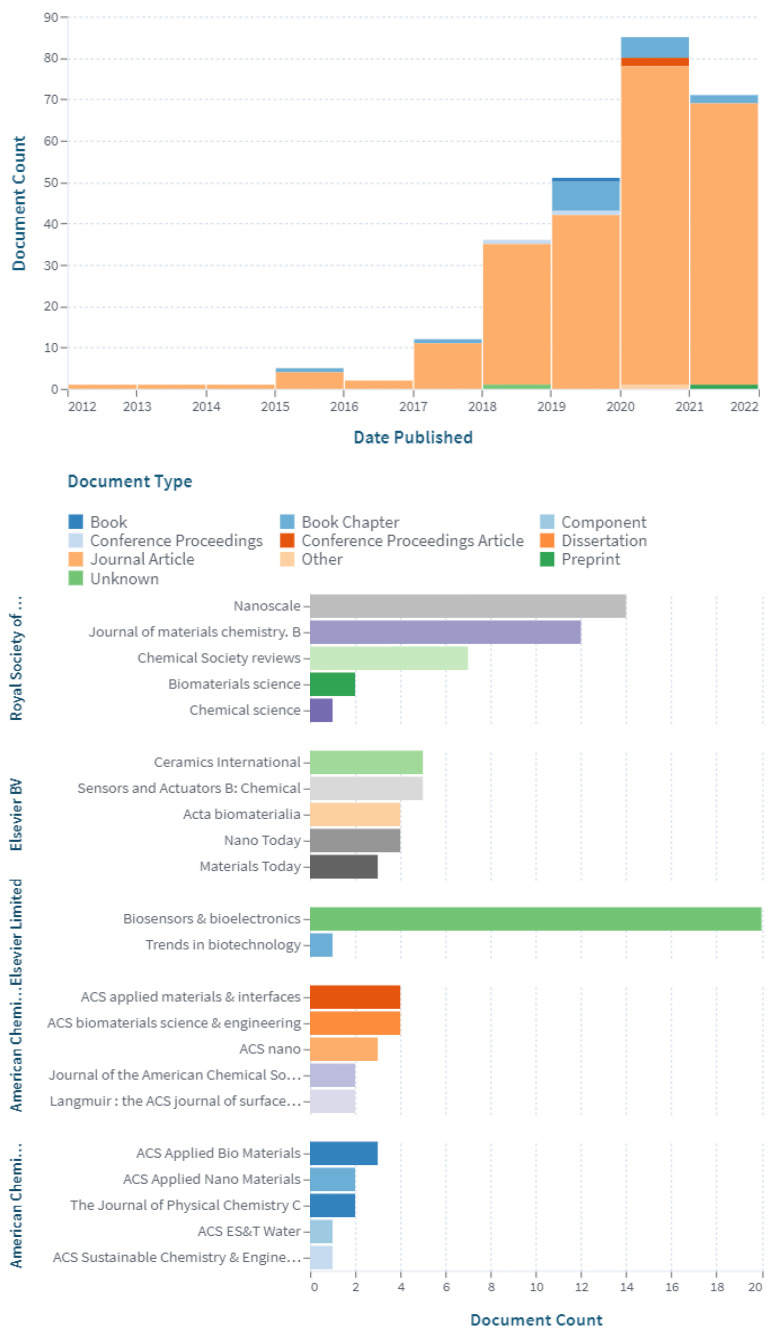
Trends related to scholarly works published using keywords “MXenes” and “biomedical” and journal details using lens.org, respectively. There is an evident increase in the number of published works each year.

Although the abovementioned primarily discusses Ti_3_C_2_T_x_ MXenes, many reports of other types of MXenes could be applicable for medical-related applications. For example, Ti_2_N MXene QDs show outstanding photothermal translation competence under laser irradiation. These Ti_2_N MXene QDs present significant biocompatibility, a photoacoustic effect, and photothermal therapy efficiency [117]. In addition, Nb_2_C MXene enveloped by S-nitrosothiol-grafted mesoporous silica with 3D-printing bioactive glass scaffolds has been developed for medical purposes. The NIR-triggered photonic hyperthermia of MXenes in the bio-window and the precisely regulated nitric oxide release could be synchronized for multi-target removal of bone tumors to improve confined osteosarcoma treatments [118]. The antibacterial activity of MXenes, especially in the form of a membrane, could endorse their potential application not only for biomedical applications but also in water treatment processes, as we explained in the previous section [110]. Many developments of MXenes are for biomedical applications. Figure 11 shows an increasing trend related to scholarly works published using the keywords “MXenes” and “biomedical” and details of the publishing journals using lens.org, respectively.

Although MXenes and MXene-based materials exhibit various properties, facile surface modification procedures are necessary to improve their biocompatibility, biodegradability, and remarkable physiological stability. Detailed investigations regarding a critical assessment of their toxicity, biocompatibility issues, solubility, dispersibility, and long-term toxicity are also crucial to exploit the biomedical application of MXenes [114]. The MXenes’ cytotoxic effects will remain a major concern before their introduction into the human physiological system. There are a limited number of studies investigating the in vitro and in vivo cytotoxicity of MXenes and MXene-based materials. Thus, conducting a thorough assessment and discussing the current prospects related to the toxicity evaluation of MXenes and MXene-based materials for pragmatic applications is critical. Table 1 is the compilation of the in vitro toxicity studies of MXenes fabricated using various techniques (with and without surface modification) for several biomedical applications.

**Figure 12:**
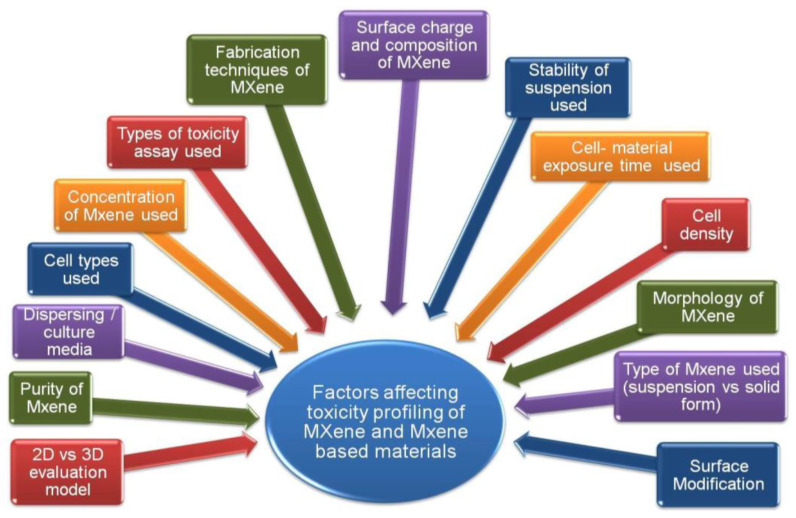
Factors affecting toxicity profiling of MXenes.

The compilation of recent studies demonstrated that MXenes are relatively non-toxic to the environment and organisms; nevertheless, additional research on MXene environmental toxicity is necessary for the future. Greater attention should be paid to the systematic assessment and adjustment of the toxicity of MXenes and MXene-based materials. For instance, for cell uptake, cytotoxicity should be thoroughly investigated in vitro and in vivo. Because MXenes and MXene-based materials have the potential to accumulate in our bodies, it is critical to understand their physiological effects. At the moment, MXenes and MXene-based materials have not been reported to interact with the human physiological system. Future work may focus on advancing and improving the limitation of gathering more in-depth toxicity evaluations in vitro and in vivo. Further research into the biocompatibility of MXenes and MXene-based nanomaterials will aid in bench-to-bedside applications, considering that they are a promising candidate for various biomedical applications. In addition, from the research compilation linked to cytotoxicity, there is a lack of agreement between the studies on the nature of MXene toxicity in cells as scientists have used a variety of cell lines (normal vs. cancer cells). Their findings suggest that the materials’ surface chemistry and functionalization can influence MXene toxicity. Additional factors that may contribute to the tested nanomaterials’ varying degrees of toxicity are listed in Figure 12.

Along with in vitro studies, extensive in vivo research has been conducted on the toxicity of MXenes and MXene-based nanomaterials. In vivo studies are preferred because they allow for the determination of nanotoxicity in an entire organism rather than just in the cells. To date, very few studies on MXene in vivo toxicity have been published using animal models, and the MXenes’ toxicity has yet to be studied in vivo, which will pave the way for additional research into developing safe biomedical applications. Additionally, it is necessary to consider the toxicity of MXene nanomaterials to aquatic animals (ecotoxicity) and plants (phytotoxicity). The in vivo, ecotoxicological, and phytotoxicological data are critical for evaluating the health and environmental effects of MXenes and MXene-based nanomaterials. Additional research is required to improve our understanding of the exposure/effect relationships; this begins with the synthesis and biological testing of the MXenes nanomaterials. Understanding ecotoxicity and phytotoxicity will aid researchers in utilizing environmentally friendly MXenes for agricultural and wastewater treatment applications that are currently in demand. Due to the scarcity of scientific reports, Table 2 summarizes the available data on the in vivo toxicity, ecotoxicity, and phytotoxicity of MXenes.

### 4.3. Application of Green MXenes in Biosensors, Chemical Sensors, and Gas Sensors

Biosensors are one of the technologies in biomedical applications. Thus, a green technology approach for biosensors is important; non-toxic biosensor devices would be safer when they are utilized as implantable biosensors, which are being explored exponentially nowadays as next-generation disease diagnosis and healing treatments. With the increasing trend of advances in science and engineering technology, implantable medical devices such as the pacemaker, cochlear implants, and real-time blood pressure sensors have been discovered and improved, and it will increasingly continue as the super-aged society develops [145]. MXenes are one of the novel materials that could be used as the main ingredient for implantable devices, such as biosensors. The MXene has been well known as a biosensing platform due to its excellent metallic conductivity, good ion-transmission behavior, distinctive biocompatibility, very large surface area, and ease of functionalization [146]. Thus, in the green technological aspect, the MXenes could be used to detect a toxic substance in the human body, whereas in the green synthesis aspect, various strategies to produce MXenes with good biocompatibility and low toxicity have been exponentially conducted; therefore, they could be more suitable for biosensors, especially implantable biosensor devices. Recently, Song et al. tried to achieve novel fluoride-free Nb_2_CT_x_ nanosheets prepared by using a facile synthesis method of electrochemical etching exfoliation [88]. Thus, by taking advantage of the rapid aluminum clearance, excellent chemical stability, and biocompatibility from the MXenes by electrochemical etching, fluoride-free Nb_2_CT_x_/acetylcholinesterase-based biosensors were successfully realized to detect phosmet, with the limit of detection being as low as 0.046 ng mL^−1^ [88]. Zhou et al. also reported an Acetylcholinesterase/chitosan–Ti_3_C_2_T_x_/GCE-based biosensor to quantify the organophosphorus pesticide, malathion [116]. Thus, an excellent biocompatibility biosensor was successfully obtained, owing to their excellent film-forming potential, the non–toxicity originating from the chitosan, as well as the high conductivity and high surface area of the MXenes, which resulted in a highly reproducible, stable, and interference-free biosensor for the quantification of the organophosphorus pesticide [116].

The MXenes could be used for many types of sensors, including chemical and gas sensors. Increasing demand for this purpose has made the MXenes become one of the emerging materials that is being developed, including by using green technology and synthesis methods. As we explained in the previous section, the biosensor is used to detect biological substances, whereas the chemical sensor is used to detect chemical substances in the form of a solid, in a solution, and as a gas. Specifically, the detection of substances in a gaseous form is interesting, and MXenes are one of the best candidates, which means that they could be used as the main ingredient for gas sensors. A gas sensor also recently has been developed for biomarker diseases in the biomedical technology field. The gas sensors’ working principle relies on the molecular adsorption/desorption process of the target gas on the MXene surface via physical and chemical processes, which results in the change of the electrical properties due to the charge transfer process [90]. Kim et al. first explored the 2D Ti_3_C_2_T_x_ MXenes as the sensing materials to detect VOCs in the parts per billion (ppb) [147]. To date, the target analytes have been extended to many harmful (NH_3_, NO, SO_2_, H_2_S, and CO) and flammable (H_2_) gases. Moreover, there are many reviews highlighting the MXenes-based materials applications as gas sensors [148,149], so the readers that are interested in more detailed discussions are referred to those reviews. In brief, pristine MXenes with O– and OH– surface terminal groups are particularly useful to sense VOCs due to the strong binding interaction between the MXenes surface and the gas molecules via hydrogen bonding [150]. Heterostructures-based MXenes were developed to enhance the gas sensing properties, and they benefit from a built-in Schottky junction and a charge-trapping mechanism [151,152]. Surface and structural engineering have been approved as alternative techniques to improve the gas sensing of MXenes towards VOCs [153,154]. Herein, we emphasize and provide the recent application of green MXenes for gas sensors. Two-dimensional V_2_CT_x_ and Ti_3_C_2_T_x_ were fabricated in various green etching solutions, such as LiF/HCl or NaF/HCl mixtures and Lewis acid (ZnCl_2_) molten salt, to tune their surface chemistry [155]. It was found that the gas-sensing properties of the 2D V_2_CT_x_ and Ti_3_C_2_T_x_ were affected by the etching solutions. V_2_CT_x_ synthesized in HF showed a positive response to methane, while the NaF/HCl mixture exhibited an excellent response to formaldehyde. Similarly, the 2D Ti_3_C_2_T_x_ etched in an NaF/HCl mixture showed a response to all of the target gases, while that which was etched in ZnCl_2_ showed a better response to ammonia, triethylamine, and toluene. The LiF/HCl mixture-etched mesoporous MXene/ZnO nanorod hybrids showed sensing responses of 346% at 200 ppb NO_2_ in ambient conditions with a response time of 17 s, and the recovery time was 24 s (at 50 ppb NO_2_) [156]. The etching route in the LiF/HCl mixtures has also been used to produce Ti_3_C_2_T_x_ that is capable of detecting H_2_S and NO_x_ gases [157,158]. Another type of MXenes, i.e., Nb_2_CT_x_ nanosheets, showed a higher sensing response towards NH_3_ after the modification with polyaniline (PANI) due to the existence of a p-n junction with hydrogen bonds [159]. The polymer modification also has improved the NO_2_ sensing performance of the Ti_3_C_2_T_x_ nanosheet [160]. From all of these results, the green approach to producing MXenes does not decrease the sensing properties of the MXenes, but it tunes them into selective sensing materials.

### 4.4. Application of the Green MXenes for Energy Harvesting

**Figure 13:**
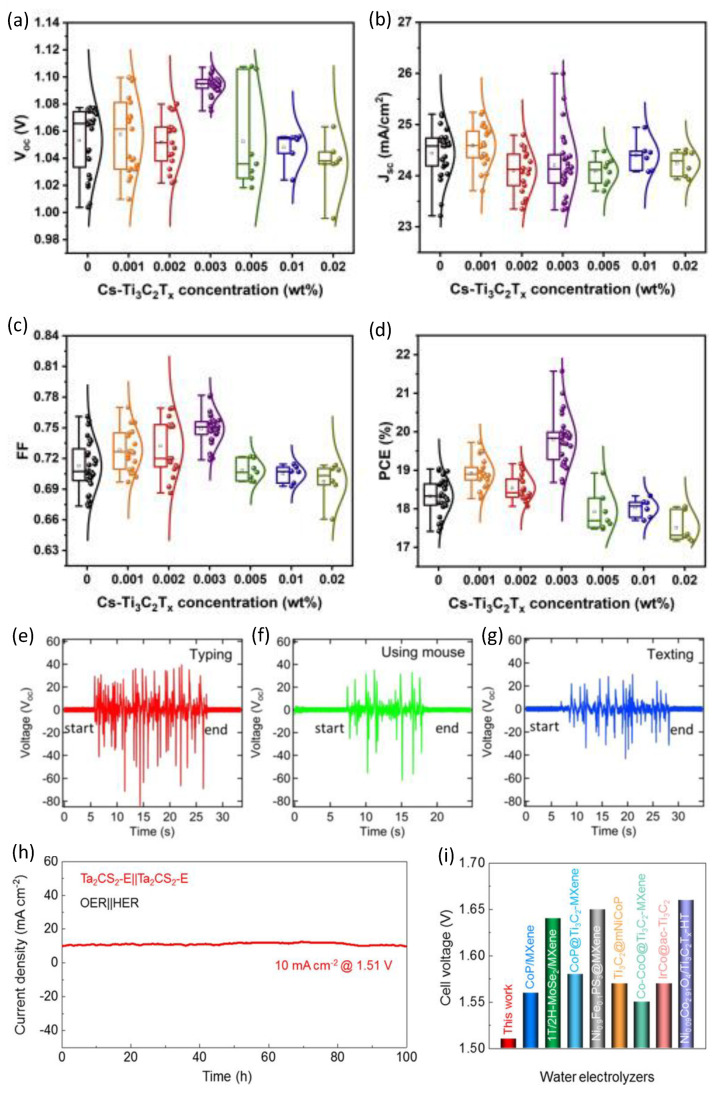
Recent applications of green MXenes in energy harvesting devices (**a**–**d**) solar cells [162], (**e**–**g**) nanogenerator [163], and (**h**–**i**) water splitting for hydrogen production [164].

MXenes could be utilized in energy harvesting and storage devices, and thus, they are created with the explicit purpose of being used in green technology. On the other hand, low-toxicity devices for energy harvesting and storage have also become important due to the need to decrease the amount of device waste and produce eco-friendly devices with a low hazardous impact on the environment. Decreasing the devices’ waste after their use would be aligned with the positive purpose of renewable energy. Therefore, the green technology approach is important in the development of energy harvesting and storage devices that are more eco-friendly. To achieve a perfect utilization of MXenes in energy harvesting, such as nanogenerators, solar cells, and hydrogen catalysts, the use of green technology in the synthesis of MXenes has become a mandatory requirement to aim for non-toxic, low-cost, and highly efficient devices-based MXenes. Mxenes have been developed for solar cell applications. According to the density functional (DFT) calculations, Zr_2_CO_2_ (Hf_2_CO_2_) and Ti_2_CO_2_ are promising donors and acceptor materials, and they possibly have a moderate band gap of 1.22 eV, and they show an excellent absorbance coefficient of 105 cm^−1^ in the visible light region [161]. Furthermore, the photocurrents of the Ti_2_CO_2_/Zr_2_CO_2_ and Ti_2_CO_2_/Hf_2_CO_2_ heterostructures are competitive with silicon-based solar cell devices, and they are predicted to own a very high power conversion efficiency of 22.74% and 19.56%, respectively [161]. Interestingly, Ti_3_C_2_T_x_ MXene-doped cesium (Cs) could act as an additive material to boost the cell efficiency of perovskite solar cells up to 21.57% as well as to obtain excellent VOCs, JSCs, and FFs, which have good thermal stability, as shown in Figure 13a–d [162]. Here, non-toxic MXenes play an essential role since lead (Pb)-based perovskite solar cells are basically toxic, whereas non-toxic additive MXenes are not only used to enhance the performance of the solar cell but they also introduce the possibility to decrease the potential toxicity in Pb-based perovskite solar cells by reducing the amount of Pb-based perovskite in the solar cells devices. Nanogenerator is one of the nanotechnology inventions that mainly utilize piezoelectric materials. Recently, Ti_3_C_2_T_x_ MXenes, which have electrically conducting properties and are also applicable triboelectrically, have more negative nanogenerators than they do polytetrafluorethylene or Teflon-based triboelectric nanogenerators [163]. Flexible MXene triboelectric nanogenerators generate high open-circuit voltages ranging from ~500 to ~650 V, and they increase the instantaneous peak power to ~0.5–0.65 mW, resulting in a power higher than that of 60 light-emitting diodes or the quick charge a 1 μF capacitor up to 50 V, and thus, they are capable of harvesting electrical power from simple muscle movements (e.g., texting) even when the device is bent by ~30°, as shown in Figure 13e–g [163]. This result shows the facile integration of flexible MXene triboelectric nanogenerators for wearable electronics, which, of course, should lower both their toxicity and production cost. Again, green technology is valid for the realization of flexible MXene triboelectric nanogenerators for wearable electronics in terms of decreasing their toxicity and cost production. Another energy conversion that includes MXenes, which promises to obtain renewable energy, is water splitting for hydrogen production. Hydrogen production from electrolyzed water plays a significant role in clean energy systems [164]. Thus, the green synthesis of MXenes is the tool to produce hydrogen, and this is important to realize this clean energy system. Previous reports have attempted to actualize this concept, for example, the optimization of the traditional synthesis routes procedure of 2D Ta_2_CS_2_, which directly use a one-step method, a more efficient synthesis, and a low-cost system [164]. In this case, Ta_2_CS_2_ is terminated in an orderly manner with S atoms. It shows excellent conductivity and electrochemical properties, plus an outstanding performance for an oxygen evolution reaction (OER) and a hydrogen evolution reaction (HER). The exfoliated Ta_2_CS_2_ (Ta_2_CS_2_-E) is found to be an exceptional bifunctional catalyst of the MXenes-based materials for overall water splitting, as shown in Figure 13h–i [164].

### 4.5. Application of the Green MXenes for Energy Storage 

**Figure 14:**
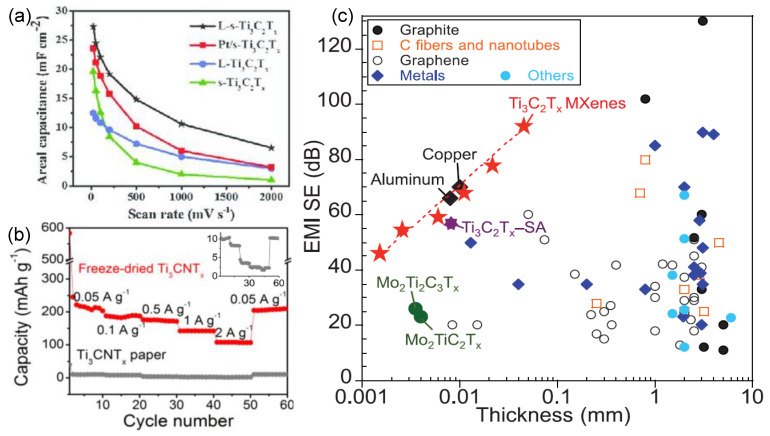
(**a**) Large Ti_3_C_2_T*_x_* (L-Ti_3_C_2_T*_x_*) and small Ti_3_C_2_T*_x_* (s-Ti_3_C_2_T*_x_*) are used as a current collector and active material [169]. (**b**) Discharge capacity of both freeze-dried and vacuum-dried Ti_3_CNT*_x_* at different cycling numbers [174]. (**c**) Comparison of EMI SE of Ti_3_C_2_T*_x_* and others at different thicknesses [176].

The use of novel energy storage concepts and devices is also one of the implementations of green technology. Recently, it was found that the green MXenes are suitable for supercapacitors, which have garnered considerable interest due to their high power density, excellent rate retention, and ultrahigh cycle life [165]. There are two types of supercapacitors that are used in charge storage mechanisms: electrical double-layer capacitors (EDLCs) and pseudocapacitors [166,167]. In recent years, green MXenes-based materials have been used as an electrode for supercapacitors. Ghidiu et al. demonstrated the use of green Ti_3_C_2_T_x_ MXenes for a supercapacitor for the first time [168]. The Ti_3_C_2_T_x_ MXenes show a volumetric capacitance of 900 F cm^−3^ at a scan rate of 2 mV s^−1^ without capacitance loss after 10,000 cycles. Peng et al. used two sizes of MXenes for solid-state micro-supercapacitors, which were prepared by green solution synthesis and solution spray coating [169]. Large Ti_3_C_2_T_x_ (L- Ti_3_C_2_T_x_) and small Ti_3_C_2_T_x_ (s- Ti_3_C_2_T_x_) are used as current collectors and active materials. The solid-state micro supercapacitor exhibits areal and volumetric capacitance values of 27 mF cm^−2^ and 357 F cm^−3^, respectively, at 20 mV s^−1^ without capacitance loss after 10,000 cycles, as shown in Figure 14a [169]. Green MXenes composites have also been demonstrated for supercapacitor applications. Yang et al. demonstrated the use of a Ti_3_C_2_/reduced graphene oxide (Ti_3_C_2_/rGO) fiber composite for flexible supercapacitors [170]. A well-aligned Ti_3_C_2_/rGO fiber composite exhibits conductivity up to 2.9 × 104 S m^−1^ with an excellent volumetric capacitance of 586.4 F cm^−3^, thereby outperforming the reported fiber-based supercapacitor. PPy-MXene-IL-mic composite films have been demonstrated to be used for flexible supercapacitors with a gravimetric capacitance of 51.85 F g^−1^ [171].

Recently, green MXenes-based materials have been demonstrated for Li-ion battery (LiBs) electrode materials. Liu et al. fabricated high-purity V_2_C by etching V_2_AlC in the presence of NaF + HCl [172]. The 90 wt.% purity of V_2_C exhibits a capacity of 260 mAh g^−1^ at 370 mA g^−1^. Wang et al. also investigated various fluoride salt etching agents in V_2_AlC [173]. Among LiF, NaF, KF, and NH_4_F, the NH_4_F-etched V_2_CT_x_ showed the highest capacity of 233 mAh g^−1^ at the current 1000 mA g^−1^ due to its large accessible active site and low resistance. Du et al. etched the Ti_3_AlCN MAX phase with LiF + HCl, which was followed by freeze drying to obtain fluffy Ti_3_CNT_x_ [174]. The freeze-dried Ti_3_CNT_x_ provides 20 times higher capacity than the vacuum-filtrated Ti_3_CNT_x_ paper does, as shown in Figure 14b [174]. This is due to it having an open structure and fewer restacking layers in the freeze-dried Ti_3_CNT_x_, thus favoring easier Li+ ion diffusion. Zhao et al. recently demonstrated the use of 3D porous Ti_3_CNT_x_ MXenes (p-MXene) for LiB [175]. The green MXene was synthesized by LiF+HCl etching, which was followed by the sulfur-template method to form p-MXenes. The p-MXene exhibits a high capacity of 314.9 mAh g^−1^ at 50 mA g^−1^ after 300 cycles, while the stacked MXene film shows a capacity of 71.3 mAh g^−1^ [175].

Further developments of the green MXenes-based electrodes focus on preventing or overcoming the re-stacking issue. Introducing other nanoparticles into the MXenes network is an effective way to solve this issue. Tian et al. etched and delaminated Ti_3_C_2_T_x_ using LiF+HCl, and then, they incorporated Si nanoparticles into the Ti_3_C_2_T_x_ network [177]. The obtained Si/Ti_3_C_2_T_x_ exhibits a small volume expansion, enhances the conductivity of the composites, and prevents the restacking of the MXene sheets and facile ion transport. As a result, the Si/Ti_3_C_2_T_x_ exhibits an exceptional electrochemical performance with a high capacity of 2118 mAh g^−1^ at 200 mA g^−1^ after 100 cycles and rate performance of 890 mAh g^−1^ at 5000 mA g^−1^. Meanwhile, the Si anode presents fast capacity decay with a capacity of <500 mAh g^−1^ at 200 mA g^−1^. The SnO_2_ quantum dots were also grown on the green Ti_3_C_2_T_x_ MXene to prevent the re-stacking of the MXene layer [178]. The composite exhibits a high capacity of 887.4 mAh g^−1^ at 50 mA g^−1^ and a stable cycle performance of 659.8 mAh g^−1^ at 100 mA g^−1^ after 100 cycles with a capacity retention of 91%. MoS_2_ [179], MoS_3_ [180], Cu_2_O [181], and Co_3_O_4_ [182] were also integrated into green Ti_3_C_2_T_x_ MXene for LiB. In addition, green MXenes-based materials have attracted massive attention as the electrode materials of other rechargeable batteries, such as Li–sulfur batteries (Li-SBs) [183,184,185], sodium-ion batteries (SIBs) [186,187,188,189], potassium-ion batteries (PIBs) [188,190,191], and multivalent-ion (i.e., Mg^2+^, Zn^2+^, Al^3+^) batteries [80,192].

### 4.6. Application of the Green MXenes for Electromagnetic Interference Shielding

Almost similar to the explanations in the previous sections of the electronics-related applications, 2D MXenes have been used for electromagnetic interference shielding (EMI) due to their high electrical conductivity, large surface area, tunable surface chemistry, and because they are lightweight [176,193,194,195]. EMI could be applicable for restricting the proliferation of electromagnetic rays inside a space or device by mounting a barrier made of a conductive material called a shield [196]. Basically, the shields are mostly made from metals, but a recent trend has appeared which involves using a coating strategy with conductive polymers, graphene, and a carbon nanotube to prepare the electro-conductive shields; it could be in textile shields [196] and several electronics, food, and vehicle packages. Research on those alternative materials has exponentially increased in recent years as green and sustainable materials replace the utilization of metals and some semiconductors for EMI, which have several health hazards [196]. The use of green materials in EMI also should be in line with a flexible, durable, lightweight, and efficient EMI shield [196]. Similarly, green technology and the green synthesis approach are important in the development of MXenes for EMI applications. The recent development of the green technology of MXenes would pave the way for green EMI applications. 

Shahzad et al. fabricated a 45-m-thick Ti_3_C_2_T_x_ film through etching in LiF+HCl, which was followed by vacuum filtration, and they used it for EMI shielding for the first time [176]. The thick Ti_3_C_2_T_x_ film exhibits 92 dB EMI shielding effectiveness (SE), which is higher than that of Mo_2_TiC_2_T_x_ and Mo_2_Ti_2_C_3_T_x_ and comparable to that of the Al and Cu metals, as shown in Figure 14c [176]. EMI shielding is also used in flexible and wearable electronics [149,195,197,198]. MXene-based composites have thus attracted significant attention in improving their electrical conductivity and mechanical strength. He et al. investigated the effect of HF and LiF/HCl etching agents on the EMI shielding properties of the Ti_3_C_2_T_x_/SiO_2_ composite [199]. The Ti_3_C_2_T_x_ obtained from LiF/HCl (u-Ti_3_C_2_T_x_) exhibits a better EMI SE than the Ti_3_C_2_T_x_ that is obtained from HF does (m-Ti_3_C_2_T_x_). This is because the u-Ti_3_C_2_T_x_ has higher electrical conductivity and a large surface area than the m-Ti_3_C_2_T_x_ does. The highly flexible Ti_3_C_2_T_x_/cellulose nanofiber (CNF) has an electrical conductivity of 1.155 S cm^−1^ and an EMI SE of 25.8 dB at 12.4 GHz, which is higher than its constituent [200]. A carbon nanotube (CNT) is incorporated into Ti_3_C_2_T_x_ aerogel to enhance the electrical conductivity and mechanical strength further [201]. The Ti_3_C_2_T_x_/CNT exhibits a high electrical conductivity of 9.43 S cm^−1^ and a superior EMI SE of 103.9 dB at a 3 mm thickness. Additionally, the layer-by-layer structure has been demonstrated to enhance the mechanical strength of EMI shielding, e.g., Ti_3_C_2_T_x_/PPy is fabricated layer-by-layer by dip coating the poly(ethyleneterephthalate) textile into Ti_3_C_2_T_x_/PPy ink several times [202]. The Ti_3_C_2_T_x_/PPy-containing textile exhibited an electrical conductivity of 1000 S m^−1^ and an SE of 90 dB at a thickness of 1.3 mm. 

## 5. Future Perspective and Summary

Due to their attractive properties, the expansion of the MXenes’ development has advanced beyond purely the academic interests. As a result, MXenes have been explored over the past decades for various emerging and broad applications such as clean energy conversions, energy storage, biomedicals, sensors, etc., but many challenges still hamper their practical relevance. Current extensive research should address the drawbacks associated with their synthesis, considering the importance of the MXenes in many other fields and their full potential in scaled-up production to achieve greener and safer methods. MXene synthesis involves multistep processes, including MAX phase synthesis and etching, which require initial exothermic reactions and the release of toxic gases during the etching process [203]. 

As a starting precursor of MXenes, the MAX phase is generally prepared by a solid-state reaction from expensive laboratory-grade elemental powders at high temperatures in an inert atmosphere. Despite the successful attempts to synthesize MXenes, the cost of the precursor MAX phase material is of great concern, and the requirement of an inert atmosphere and an additional treatment to obtain a fine powder contributes to the increasing operating costs. Therefore, using cheap materials and cost-efficient methods to produce MAX phases will suppress the associated costs and, eventually, the overall production cost of MXenes synthesis. A study reported that MXenes could be successfully synthesized from the MAX phases that utilize inexpensive metal oxide as the transition metal source [204]. Other reports that also shed light on the MXenes’ development found that TiO_2_, recycled aluminum scrap, and carbon recovered from waste tires could be the low-cost and environmentally friendly precursors of the Ti_3_AlC_2_ MAX phase to synthesize the Ti_3_C_2_T_x_ MXenes [9].

In synthesizing the MAX phase, the need for high-temperature conditions results in an energy-intensive process. Without renewable electricity, this synthesis process consumes a lot of fossil-based energy and contributes to releasing CO_2_ emissions into the atmosphere, potentially accelerating global warming and climate change. To overcome this issue, alternative methods have been pursued to produce MAX in milder conditions with a comparable yield and high purity, such as molten salts and sol-gel techniques. The molten salt shielded synthesis method was recently carried out in an air atmosphere, at a lower temperature, without an additional milling step, and this could possibly be adopted to large-scale MAX phase powder [205,206]. This alternative can significantly encourage more experimental research on producing MXenes in a more energy-efficient way.

In a further step, most of the MXenes have been prepared by selectively etching the A element from the MAX phase, with the etchants being used. Among the available enchants, direct HF is the most widely used one, and it is suitable to synthesize a broad variety of MXenes from the corresponding MAX phases despite its high toxicity. As the extreme toxicity of HF is a crucial concern to this strategy, proper safety precautions must be taken while handling HF or decanting HF waste [203]. Other methods introducing less toxic chemicals, such as in situ HF etching from the highly concentrated mixture of LiF and HCl, have been introduced despite the waste solutions containing F potentially leaking into the water environment. It is well known that F is the primary source of skeletal fluorosis. Moreover, the remaining F on the MXenes’ surface may decrease the device’s performance when it is based on MXenes. The exploration of fluoride-free and environmentally friendly methods should be devoted to identifying sought-after alternatives for HF-containing chemicals. 

In this pursuit, while the choice of initiating the MAX phase is non-negotiable for the synthesis of 2D MXenes, the alternative synthesis solvents are frequently adjustable. Under critical conditions, more mild etchants such as NaOH, NaBF_4_, and HCl have the potential to etch the MAX phase favorably (hydrothermal and solvothermal ones). This condition produces MXenes that are susceptible to surface oxidation, which is useful for constructing in situ nanocomposite heterostructures, especially for energy storage and conversions. Other green solvents include molten salts and electrochemical etching with the aid of ionic liquid. In the case of molten salts, the inorganic salts melted at high temperatures will eliminate the A layer and prevent surface oxidation, as the MAX phase is buried beneath the molten salts. Electrochemical etching is favorable if the MXenes are to be used for electrochemically related applications such as catalysis, energy storage, or applications of a similar nature because MAX is deposited on a conductive substrate and etched under anodic conditions, resulting in the deposition of the 2D MXenes flakes directly on the substrate. The electrolytes for electrochemical etching are typically NaOH or HCl, but room-temperature ionic liquids (RTIL), which are considered to be environmentally friendly solvents, have also been used. However, the careful selection of an RTIL is essential for their use in green chemistry as some RTILs contain F ions that can degrade into HF over time [168]. 

While the vast majority of MXene synthesis research has been focused on finding greener etching solvents, the number of biological substances that are used to stabilize and exfoliate 2D MXenes into the single-layer form will increase in the future. The morphology features (size and shape) of the MXenes can be controlled into QDs, 1D, 2D, and 3D structures, where some forms, such as QDs, 1D, and 2D shapes, are difficult to stabilize as a colloid. To this end, bio-inspired molecules such as protein and enzymes may be leveraged as stabilizing agents to synthesize shape-controlled QDs, 1D, 2D, and 3D MXenes, which have been used in many nanomaterial synthesis processes, such as Ag, Au, etc. Although these biomolecules are in their infancy, the biological substances can offer numerous advantages, such as the waste solvents are easy to handle, they are abundant in nature, and they have a simple reactor design. 

Further investment should be addressed in integrating the MXenes into living biological systems, muddled biological mechanisms, and their potential toxicity since this knowledge is lacking compared to that of other applications. MXene-based biosensors, contrast agents in cancer therapy, and antibacterial agents are a few examples of how MXenes can be biocompatible with living tissues. It is noteworthy that bio-related applications of MXenes are at an early stage compared to other biocompatible materials. Thus, expanding the practicability of the MXenes in this vital field should be encouraged. 

Some highlights of the green synthesis approaches owing to them having several advantages are: they are less toxic as they avoid the use of toxic etching agents; they have a facile synthesis as they involve a relatively more straightforward experimental process; they have low-cost processes as they eliminate the inclusion of pieces of equipment; they have feasible commercialization potential as the advantages will support their commercialization. Even though MXenes are promising and attractive materials, significant research efforts to optimize the green and safer synthesis and achieve cost- and energy-efficient techniques are critical to realizing their practical applications. Ongoing challenges and opportunities exist for the breakthrough toward their development.

## Figures and Tables

**Table 1:** Compilation of MXenes and MXene-based materials preparation techniques and their toxicity evaluation.

Type of MXene/MXene-Based Materials	Fabrication Method	Toxicity Test Approach	Toxicity Test Assay	Findings	References
MnO_x_/ Ta_4_C_3_-SP composite nanosheets	Two-step exfoliation	In vitro 4T1 (mouse mammary tumor) cell line	CCK-8	MnO_x_/Ta_4_C_3-_SP does not affect 4T1 cell survival even at high concentrations for 24 and 28 h, indicating excellent cytocompatibility.	[119]
Ti_3_C_2_, Ti_3_C_2_-SP nanosheets	Chemical exfoliation and intercalation	In vitro 4T1 (mouse mammary tumor) cell line	CCK-8	The toxicity of Ti_3_C_2_-SP to 4T1 breast cancer cells is assessed for 24 and 48 h and revealed no effect on the survival of 4T1 cells.	[120]
Ti_3_C_2_	Etching	In vitro HCT-116 (human colorectal carcinoma cell line) and A2780 (ovarian cancer cell line)	MTT assay	The cytotoxicity of Ti_3_C_2_ nanosheets is assessed using an MTT assay, indicating that cytotoxicity is dose-dependent and cell-type-dependent.	[121]
Ti_3_AlC_2_, Ti_3_SiC_2_, and Ti_2_AlN	Hot pressing and in situ sintering	In vitro MC3T3-E1 (mouse pre-osteoblast) and L929 (mouse fibroblast) cell lines	MTS assay	Compared to commercial Ti–6Al–4V alloy and pure Ti, all phases are not toxic to pre-osteoblasts and fibroblasts cell lines. Ti_2_AlN performed best in the MAX phases for cell proliferation and differentiation.	[122]
Ti_3_C_2_	Self-propagation high-temperature synthesis (SHS)	In vitro A549 (human alveolar basal epithelial cells), MRC-5 (human normal lung cells), A375 (human skin malignant melanoma cells), and HaCaT (human immortalized keratinocytes)	MTT assay	Toxicity increases with the concentration of MXene. The results reveal that the toxic effects are higher against cancerous (A549 and A375) cells than they are against normal (MRC-5 and HaCaT) cells.	[123]
Ti_3_C_2_ QDs	Etching-assisted exfoliation method, mechanical force-assisted liquid exfoliation	In vitro HeLa (human cervical cancer), MCF-7 (human breast cancer), U251 (human malignant glioblastoma), and HEK 293 (human embryonic kidney) cells	MTT assay	MXene QDs show no cytotoxicity to all tested cell lines (HeLa, MCF-7, U251, and HEK 293), even at the highest concentration of 100 ppm. Data obtained show excellent biocompatibility and indicate high clinical potential application.	[37]
Ti_3_C_2_-based MXene Integrated Cellulose Hydrogels	Etching and exfoliation	In vitro HepA1-6 (mouse hepatoma cells), SMMC-7721 and HepG2 (human hepatocellular carcinoma cells), U-118MG (human glioblastoma cells), and U-251MG (human astroglioma cells)	CCK-8 assay	The Ti_3_C_2_-based MXene integrated cellulose hydrogel exhibits excellent cellular biocompatibility as the addition of Ti_3_C_2_ MXene nanosheets to hydrogels do not affect cell viability regardless of MXene concentration. MXene integration into the hydrogel also reduces in vitro toxicity compared to dispersed MXenes.	[124]
Ti_3_C_2_-SP ultrathin nanosheets	Two-step exfoliation	In vitro 4T1 (mouse mammary tumor) cell line	CCK-8 assay	In vitro cytotoxicity of Ti_3_C_2_-SP using CCK-8 assay indicates that 4T1 cells treated with Ti_3_C_2_-SP at various concentrations for 12, 24, and 48 h have no obvious cytotoxicity, even at the highest concentration of 600 µg mL^−1^.	[111]
Ta_4_C_3_-IONP-SP	Liquid exfoliation	In vitro 4T1 (mouse mammary tumor) cell line	CCK-8 assay	In breast cancer cell lines, 4T1 cells exposed to various concentrations of Ta_4_C_3_-IONP-SP indicate no detectable cytotoxicity, even at the highest concentration of 200 ppm for 24 h.	[125]
TiC, Ti_2_AlC, and Ti_3_AlC_2_	Etching, etching coupled with intercalation	In vitro HeLa (cervical cancer cells) and MSU1.1 (normal fibroblasts)	WST-1 assay, Live/Dead assay	TiC, Ti_2_AlC, and Ti_3_AlC_2_ at concentrations (≥400 μg/mL) induce a significant cytotoxic effect in HeLa cells, while MSU1.1 cells demonstrate slight cytotoxic behavior for all Ti_3_C_2_T_x_ forms. The cytotoxicity is also cell-type-dependent, with cancer cells exhibiting greater toxicity than normal cells do.	[126]
Ti_2_C-PEG	Etching	In vitro A375 (human skin malignant melanoma cells), HaCaT (human immortalized keratinocytes), MCF-7 (human breast cancer cells), and MCF-10A (normal human mammary epithelial cells)	MTT assay	Normal (nonmalignant) cells retain 70% viability after 24 h of exposure to Ti_2_C-PEG MXene flakes, indicating that delaminated Ti_2_C-PEG is biocompatible. Ti_2_C-PEG is cytotoxic to a malignant breast cancer cell line. In comparison, at all concentrations tested, MXenes have a negligible effect on the viability of skin cancer cells. After 48 h of exposure, the viability of each cell line decreases significantly, indicating that Ti_2_C flakes are toxic in a time-dependent manner.	[127]
Ti_3_C_2_T_x_-based nanocomposites (Au/MXene and Au/Fe_3_O_4_/MXene)	In situ reduction of tetrachloroauric acid using NaBH_4_	In vitro MCF7 (human breast cancer/adenocarcinoma cell line)	Alamar Blue assay	Both new composites inhibit human breast cancer cells MCF7 in vitro in a dose-dependent manner. Even at high concentrations, no cytotoxicity is observed, indicating the composites’ high biocompatibility.	[128]
Multilayered Ti_3_C_2_T_x_	Etching	In vitro MC3T3-E1 (pre-osteoblast cell line)	EdU-488 assay, Live/dead double staining	Cells cultured on MXene films show no evidence of cytotoxicity as assessed using the EdU assay. Ti_3_C_2_T_x_ MXene demonstrates favorable cytocompatibility, cell spreading, and proliferation, proving that Ti_3_C_2_T_x_ MXene is extremely biocompatible in vitro.	[129]
Ti_3_C_2_ and Ti_2_C	Self-propagation high-temperature synthesis (SHS)	In vitro A375 (Human skin malignant melanoma cells), HaCaT (human immortalized keratinocytes), MCF-7 (human breast cancer cells), and MCF-10A (Mammary epithelial cells)	MTT assay	Biocompatibility of 2D Ti_3_C_2_ and Ti_2_C MXenes enhances post-modification with collagen compared to cultures exposed to pure 2D MXenes. Additionally, the reduction in cell viability is negligible across a broad tested concentration range.	[130]
Ti_3_C_2_	LiF/HCl delamination method followed by post-treatment using probe sonication and thermal oxidation	In vitro MCF-10A (human epithelial breast), MCF-7 (breast cancer), HaCaT (human immortalized keratinocytes), A375 (human malignant melanoma)	MTT assay	Selective cytotoxicity is against tumor cells compared to normal cells, even at high concentrations of tested materials. The most cytotoxic effect is seen in samples that are thermally oxidized. The thermally oxidized samples are also cytotoxic to all cancer cell lines.	[131]
Nb_2_C quantum dots	Hydrothermal method and nitrogen and sulfur co-doping	In vitro Caco-2 (human colorectal adenocarcinoma) cells	CCK-8 assay	Cellular viability is completely lost at the highest concentration of 20 mg/mL. None of the concentrations tested at lower concentrations of less than 10 mg/mL reduce cellular viability.	[132]
Non-oxidized MXene-Ti_3_C_2_T_x_ quantum dots (NMQDs-Ti_3_C_2_T_x_)	Micro-explosion method	In vitro HeLa (cervical cancer cell), MCF-7 (breast cancer cell), and normal ADSCs (Adipose-derived stem cells)	CCK-8 assay	NMQDs-Ti_3_C_2_T_x_ selectively kills cancer cells.	[133]
Ti_3_C_2_	Etching	In vitro Primary mouse and derived NSCs (neural stem cells)	CCK-8 assay	MXene biocompatibility in the nervous system and NSC-Ti_3_C_2_ nanosheet interactions are evaluated. Ti_3_C_2_ shows dose-dependent toxicity towards primary NSCs and differentiated NSCs. Ti_3_C_2_ nanosheets cause apoptosis, membrane disruption, stress, and inflammation at higher concentrations.	[134]
Ti_3_C_2_ and Nb_2_C quantum dots	Acid reflux followed by hydrothermal method	In vitro HUVECs (human umbilical vein endothelial cells)	CCK-8 assay	Ti_3_C_2_ QDs are more toxic than Nb_2_C QDs are, and HUVEC toxicity is expected owing to the metal ions. The morphology of QDs-based also contributes to cytotoxicity.	[135]
Ti_3_C_2_	Intercalation	In vitro hMSCs, (human mesenchymal stem cells)	MTS assay	MXene concentrations >50 µg/mL are cytotoxic over 7 days of the exposure period.	[136]
V_2_AlC, m-V_2_CT_z_, pristine and oxidized s-V_2_CT_z_	Etching	In vitro A375 (human skin malignant melanoma cells), HaCaT (human immortalized keratinocytes)	MTT assay	Immortalized keratinocyte (HaCaT) and malignant melanomas (A375) human cell lines are exposed to s-V_2_CT_z_-ox24 and s- V_2_CT_z_ -ox48 flakes. At concentrations above 50 µg/mL, only 50% of cells are viable, suggesting oxidized V_2_CT_z_ increases cytotoxicity toward human cells.	[137]
Ti_3_C_2_, Ti_3_C_2_-PEG, Ti_3_C_2_-PPG	Etching	In vitro MCF-7 (human breast cancer cells), MCF-10A (normal human mammary epithelial cells), A375 (human skin malignant melanoma cells), and HaCaT (human immortalized keratinocytes)	MTT assay	MXene cytotoxicity is directly related to cell line type, with HaCaT being the least toxic and A375 being the most. PEGylated and PPGylated MXenes show increased toxicity to both normal and cancerous cell lines.	[138]
Ti_3_C_2_T*_x_* -PVA Free-standing Ti_3_C_2_T*_x_* and PVA-Ti_3_C_2_T*_x_* films	Etching and vacuum-assisted filtration	In vitro HUVECs (human umbilical vein endothelial cells)	Live/dead assay	There is no evidence of cytotoxicity based on the assay. More live cells (green) than dead cells (red) are observed, indicating that most HUVECs are healthy for 7 days with viability of 99.8%. PVA-MXene’s in vitro biocompatibility is critical for biomedical applications.	[139]
Ti_2_CT_X_	Etching	In vitro HeLa (cervical cancer cell)	MTT assay, calcein-AM staining	Two-dimensional and three-dimensional model HeLa cells are used to assess the cytotoxicity of Ti_2_CT_x_ MXenes. Two-dimensional culture system shows that cells’ viability decreased to half with increasing Ti_2_CT_x_ MXenes concentration. Three-dimensional spheroid better mimics real physiological conditions, so cells are highly viable after 15 days of culture at the lowest concentration of Ti_2_CT_x_ MXenes, and some cells are found non-viable when concentration increases to 500 μg/mL.	[140]
Ti_3_C_2_T_x_-UHAPNWs	Etching	In vitro MC3T3-E1 (pre-osteoblast cell line)	Live/dead double staining	Staining for live/dead cells demonstrates no discernible difference between cells seeded on glass and UHAPNWs/MXene, indicating that the films are non-toxic. MC3T3-E1 cells are tightly attached to the surface and appear flattened, spread more rapidly, and even confluence. Osteogenic differentiation in MC3T3-E1 cultures grown on Ti_3_C_2_T_x_ nanosheet also increases.	[141]

**Table 2:** In vivo, ecotoxicity, and phytotoxicity of MXenes and MXene-based nanomaterials.

Type of MXene/MXene-Based Materials	Fabrication Method	Model of the Living Organism Used	Findings	References
Ti_3_C_2_ QDs	Etching-assisted exfoliation method, mechanical force-assisted liquid exfoliation	Balb/c mice	There are no obvious signs of abnormal mouse weight, diet, or activity. H and E staining reveals no obvious tissue or organ damage. MXene QDs are not toxic to mice at the dosages examined, which could be attributed to the synthesis using ultrasonication rather than to the toxic organic solvents and chemicals, which ensures the safe use of MXene QDs in medicine.	[37]
MnO_x_/^breakTa_4_C_3_-SP composite nanosheets	Two-step exfoliation	Balb/c mice	When they are used as intended, the MnO_x_/ Ta_4_C_3_-SP composite nanosheets are highly biocompatible and biosafe. The authors use a rational chemical composition (Ta_4_C_3_) and surface engineering for functionalization (MnOx integration). After 60 days, mice treated with MnO_x_/ Ta_4_C_3_-SP remains healthy with no evidence of tumor recurrence, confirming the high therapeutic efficacy of MnO_x_/Ta_4_C_3_-SP composite nanosheets in vivo.	[119]
Ti_3_C_2_	Etching	Athymic nude mice	Histopathologically, H and E staining reveals the formation of small nuclei in the tumors of DOX and Ti_3_C_2_-DOX-treated mice, indicating cell apoptosis. The low dose and the stimuli-responsive drug release prevented significant organ damage. There are no significant morphology and pathology changes in the treated mice.	[121]
Ti_3_C_2_-SP	Two-step exfoliation	Balb/c mice	No significant morphology and pathology changes in major organs of treated mice suggest that Ti_3_C_2_-SP nanosheets have no obvious acute toxicity and side effects. Intravenous Ti_3_C_2_-SP injection can be easily excreted from the body via urine and feces.	[111]
Ta_4_C_3_-IONP-SP	Liquid exfoliation	BALB/c mice	The in vivo biocompatibility of Ta_4_C_3_-IONP-SP composite nanosheets are assessed in four groups of healthy Kunming mice (5, 10, and 20 mg kg^−1^). Ta_4_C_3_-IONP-SP is given intravenously for a month. None of the mice lose weight or die. The experimental mice’s main organ H and E staining sections show no significant damage or acute inflammation compared to the control group. Based on preliminary biocompatibility data, Ta_4_C_3_-IONP-SP composite nanosheets are safe to use in clinical settings, especially when used to guide hyperthermia and ablation of breast tumors in vivo.	[125]
Ti_3_C_2_T_x_-based nanocomposites (Au/MXene and Au/Fe_3_O_4_/MXene)	In situ reduction of tetrachloroauric acid using NaBH4	Zebrafish embryo	Au/MXene and Au/Fe_3_O_4_/MXene-treated embryo groups normally develop. Data obtained indicate that Au/MXene and Au/MXene have almost no acute toxic or teratogenic effect on zebrafish embryos. Au/MXene and Au/Fe_3_O_4_/MXene are biocompatible and safe when compared to pure MXene.	[128]
Multilayered Ti_3_C_2_T_x_	Etching	Sprague Dawley rats	Inflammatory reactions are not seen in nearly all defect spaces under MXene films. The regenerated bone is flat and uniform with an osteoid collagen fiber separated MXene films from the osteoid tissue. The MXene group’s new bone volume is much larger than the control group’s one is. The host tissue response to Ti_3_C_2_T_x_ MXene films confirms their safety and high biocompatibility in vivo. Morphologically, MXene films promote early osteogenesis, mineralization, and bone regeneration in rats. These findings show Ti_3_C_2_T_x_ MXene is highly biocompatible.	[129]
Non-oxidized MXene- Ti_3_C_2_T_x_ quantum dots (NMQDs-Ti_3_C_2_T_x_)	Micro-explosion method	BALB/c mice	The NMQDs- Ti_3_C_2_T_x_ killing tumor spreads from within. The H and E staining reveals significant destruction of tumor cells, and TUNEL images show increased cell apoptosis. NMQDs- Ti_3_C_2_T_x_ does not cause obvious pathological changes in the tissues of the heart, liver, spleen, lung, and kidney, demonstrating its high biocompatibility in vivo.	[133]
Ti₃C₂T_x_	Etching	Avian embryos	The toxicity of MXene nanosheets on early embryonic development and angiogenesis is evaluated using 3- and 5-day-old avian embryos as a model. Forty-six percent of MXene-exposed embryos die 1–5 days after exposure, indicating MXene may cause early embryonic death. After 5 days of exposure to MXene, the inhibition of embryonic chorioallantoic membrane angiogenesis is detected.	[142]
Ti_3_C_2_T_x_-UHAPNWs	Etching	Sprague Dawley rats	MXene films can promote osteogenic differentiation and bone formation, which accelerate bone regeneration in a calvarial bone defect in rats. The fabricated nanocomposite films’ novel structure and surface morphology result in excellent mechanical properties, biocompatibility, and osteoinductivity. MXene and MXene-based nanocomposites are critical in biomedical applications, particularly bone regeneration.	[141]
Ti_3_C_2_T_x_	Delaminating and ultrasonication	Zebrafish embryo	No teratogenic effects on zebrafish embryos are observed at 100 µM, where Ti_3_C_2_T_x_ is in a homogeneous solution. High concentrations of Ti_3_C_2_T_x_ (>100 µM) have a minimal teratogenic effect on embryos. The mortality effect may be due to the Ti_3_C_2_T_x_ aggregation, and the embryos cannot tolerate it. Ti_3_C_2_T_x_ is completely non-toxic to aquatic life. It may be necessary to assess the toxicity of Ti_3_C_2_T_x_ (MXene) nanosheets in aquatic ecosystems other than zebrafishes.	[143]
Ti_3_C_2_ MXene with ceramic oxide and noble metal	SHS technique with a local ignition system	Green algae Desmodesmus quadricauda	Pristine Ti_3_C_2_ MXene stimulates algal growth at low concentrations. The effect of nano component concentration on stimulation decreases. Ecotoxicity against algae depends not only on concentration but also on modification type (ceramic oxide and noble metal used).	[144]
Ti_3_C_2_ MXene with ceramic oxide and noble metal	SHS technique with a local ignition system	Seed of sorghum (Sorghum saccharatum) and charlock (Sinapis alba)	Modification of Ti_3_C_2_ MXene is used to study its phytotoxicity. It becomes more phytotoxic when it is modified with SiO_2_/Ag or SiO_2_/Pd is added, but not when Ti_3_C_2_/Al_2_O_3_/Ag is added. The modified nanocomposites have a lower inhibitory effect on germination than Ti_3_C_2_ MXene. When different nanoparticles are added to pure Ti_3_C_2_ MXene, its phytotoxic properties can change.	[144]

## Data Availability

The study did not report any data.
